# Protein DEK and DTA Aptamers: Insight Into the Interaction Mechanisms and the Computational Aptamer Design

**DOI:** 10.3389/fmolb.2022.946480

**Published:** 2022-07-19

**Authors:** Lijun Dai, Jiangnan Zhang, Xiaonan Wang, Xiaoyue Yang, Feng Pan, Longhua Yang, Yongxing Zhao

**Affiliations:** ^1^ School of Pharmaceutical Sciences & Key Laboratory of Advanced Drug Preparation Technologies, Ministry of Education, Zhengzhou University, Zhengzhou, China; ^2^ Department of Statistics, Florida State University, Tallahassee, FL, United States; ^3^ Key Laboratory of Targeting Therapy and Diagnosis for Critical Diseases, Zhengzhou, China

**Keywords:** DEK, 2'-OCH_3_-modification, interaction mechanisms, stability and affinity, aptamer design

## Abstract

By blocking the DEK protein, DEK-targeted aptamers (DTAs) can reduce the formation of neutrophil extracellular traps (NETs) to reveal a strong anti-inflammatory efficacy in rheumatoid arthritis. However, the poor stability of DTA has greatly limited its clinical application. Thus, in order to design an aptamer with better stability, DTA was modified by methoxy groups (DTA_OMe) and then the exact DEK–DTA interaction mechanisms were explored through theoretical calculations. The corresponding 2′-OCH_3_-modified nucleotide force field was established and the molecular dynamics (MD) simulations were performed. It was proved that the 2′-OCH_3_-modification could definitely enhance the stability of DTA on the premise of comparative affinity. Furthermore, the electrostatic interaction contributed the most to the binding of DEK–DTA, which was the primary interaction to maintain stability, in addition to the non-specific interactions between positively-charged residues (e.g., Lys and Arg) of DEK and the negatively-charged phosphate backbone of aptamers. The H-bond network analysis reminded that eight bases could be mutated to probably enhance the affinity of DTA_OMe. Therein, replacing the 29th base from cytosine to thymine of DTA_OMe was theoretically confirmed to be with the best affinity and even better stability. These research studies imply to be a promising new aptamer design strategy for the treatment of inflammatory arthritis.

## 1 Introduction

Rheumatoid arthritis (RA) and juvenile idiopathic arthritis (JIA) are two classical inflammatory arthritides, both of which have been found to cause substantial disability in adults and children. Therein, RA is a common, chronic, and systemic autoimmune disease with a global incidence of about 0.25–1% (Alhajeri, et al., 2019), which can cause severely problematic pathologies, such as joint damage, disability, decreased quality of life (Scott, et al., 2010), and even death. JIA is a heterogeneous group of diseases, characterized by arthritis of an unknown origin with onset mainly before 16 years of age (Prakken, et al., 2011). It is the most common childhood chronic rheumatic disease and causes much disability, which poses a serious threat to children’s physical and mental health. DEK was first identified as a fusion protein in patients with a subtype of acute myelogenous leukemia (AML) (Ishida, et al., 2020). Subsequently, DEK was found to be the target of auto-antibodies in several autoimmune diseases, such as JIA (Dong, et al., 2000), systemic lupus erythematosus (Dong, et al., 1998; Dong, et al., 2000), sarcoidosis (Dong, et al., 1998; Dong, et al., 2000), and systemic sclerosis (Dong, et al., 2000). Several studies have suggested that DEK is crucial for neutrophils to form NETs, structures composed of DNA, histones, and antimicrobial factors that have been reported to play a part in the pathogenesis of inflammatory and autoimmune diseases, including RA (Brinkmann, et al., 2004; Khandpur, et al., 2013). Thus, DEK-targeting therapy can greatly impair the ability of neutrophils to form NETs and reduce joint inflammation (Mor-Vaknin, et al., 2017).

Nucleic acid aptamers obtained by the Systematic Evolution of Ligands by Exponential Enrichment (SELEX) technology are short, single-stranded (ss) DNA or RNA with high specificity and affinity (Kinghorn, et al., 2017) and are DNA analogs to antibody-mimetic proteins (Hu, et al., 2019). However, the traditional SELEX technology has the disadvantages of long cycles and high cost when screening aptamers. Fortunately, because of the ability to fold into 3D scaffolds, aptamers can specifically recognize and bind to their cognate targets through unique three-dimensional structures (Zhou and Rossi, 2017), which may provide a good strategy for the treatment of some debilitating chronic diseases, such as RA and JIA. Especially, Mor-Vaknin et al. screened out an anti-DEK aptamer that is a single-stranded DNA (DTA, sequence: 5′ GGG GTT AAA TAT TCC CAC ATT GCC TGC GCC AGT ACA AAT AG 3′) with 41 bases and a high affinity for recombinant DEK proteins (Mor-Vaknin, et al., 2017). It is deemed to have a potential role in inhibiting the formation of NETs and inflammatory arthritis. Combining these arguments, some researchers believe that DEK is a potential therapeutic target, and that DTA may be a promising therapeutic molecule for inflammatory arthritis (Cao, et al., 2021).

Due to the special nucleic acid nature, the DTA aptamer can offer many advantages over antibodies for targeted diagnosis and therapeutics, such as low cost, uniform synthesis, and easy modification (Wang, et al., 2019). Despite these advantages, the poor stability of DTA has dramatically limited its application *in vivo*, because of the easy degradability by nucleases (Röthlisberger and Hollenstein, 2018). A common solution to this problem in clinical studies nowadays is the chemical modifications of the aptamer. A typical strategy is to replace the 2′-position of deoxyribose with a fluoro-(F) or with amino-(NH_2_) and methoxy-(OCH_3_) groups (Zhou and Rossi, 2017). Note that the 2′-NH_2_-modification is rarely used due to modest coupling efficiencies during solid-phase synthesis and its preference for the C2′-endo (“DNA-like”) ribose conformation (Röthlisberger and Hollenstein, 2018). But, Cummins et al. reported that all oligoribonucleotides modified by 2′-OCH_3_ appear to be resistant to nuclease degradation (Cummins, et al., 1995). Therefore, it is a good choice to modify DTA with methoxyl groups to improve its stability. So far, three aptamers modified by 2′-OCH_3_ have been designated and applied, including one approved drug (Macugen®/pegaptanib), and two in late-stage development (Zimura®/ACR1905 and Fovista®/E10030) (Drolet, et al., 2016).

**FIGURE 1:**
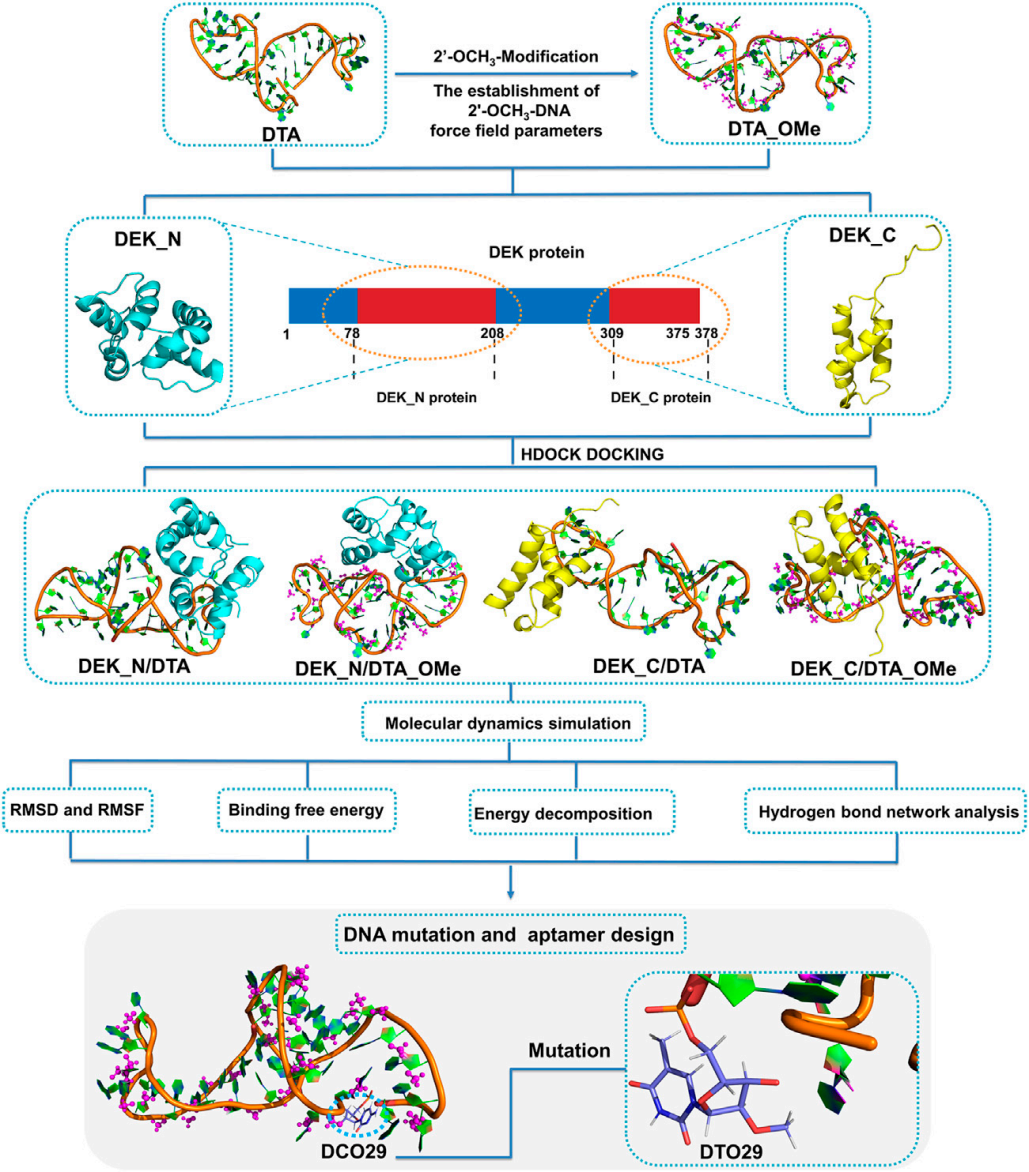
Schematic illustration of the interaction mechanisms and the new aptamer design. DEK_N and DEK_C respectively represent the N-terminal and C-terminal structural domains of the human DEK protein. DTA_OMe is obtained by modifying the 2′-position of all deoxyribose on the DTA with methoxy groups. Four complex models (DEK_N/DTA, DEK_N/DTA_OMe, DEK_C/DTA, and DEK_C/DTA_OMe) got through the cross docking between these two terminal proteins and the two aptamers.

In order to design the DEK-targeted aptamers with better stability and affinity, as shown in Figure 1, four protein–DNA complex models were constructed through docking, and MD simulations, binding free energy calculations, energy decomposition, and the hydrogen bond network analysis were applied to study the detailed interaction mechanisms of DEK–DTA/DTA_OMe. Then, based on mechanism studies, we performed a series of virtual base mutations on DTA and DTA_OMe, and found a mutant with better stability and affinity after replacing the 29th base from cytosine to thymine of DTA_OMe. These works laid a solid theoretical foundation for understanding the DEK–DTA interaction mechanism in depth, and provide a promising computational design strategy of the aptamers for the treatment of inflammatory arthritis and autoimmune diseases.

## 2 Materials and Methods

### 2.1 Structural Preparation

A human DEK protein consisting of 375 amino acids, has two functional DNA-binding domains,a central SAP/SAF box DNA-binding domain (residue 149-183), and an additional C-terminal DNA-binding region (residue 270-350) that partially overlaps with a multimerization domain or phosphorylation sites (Waldmann, et al., 2004; Böhm, et al., 2005). Now, only the DEK_N and DEK_C crystal structures are obtained by the solution NMR method instead of the full-length DEK protein structure. For structures solved by NMR, which contain multiple conformations of the same complex, the one with the lowest potential energy (usually the first) was selected and then used (Bissaro, et al., 2020). Opportunely, these two resolved structural domains (N-terminal PDB ID: 2JX3; C-terminal PDB ID: 1Q1V) (Devany, et al., 2004; Devany, et al., 2008) are overlapped with the reported two DNA-binding domains (Waldmann, et al., 2004), so utilizing these two terminal domains as target proteins are deemed a reasonable strategy in our following study.

**FIGURE 2:**
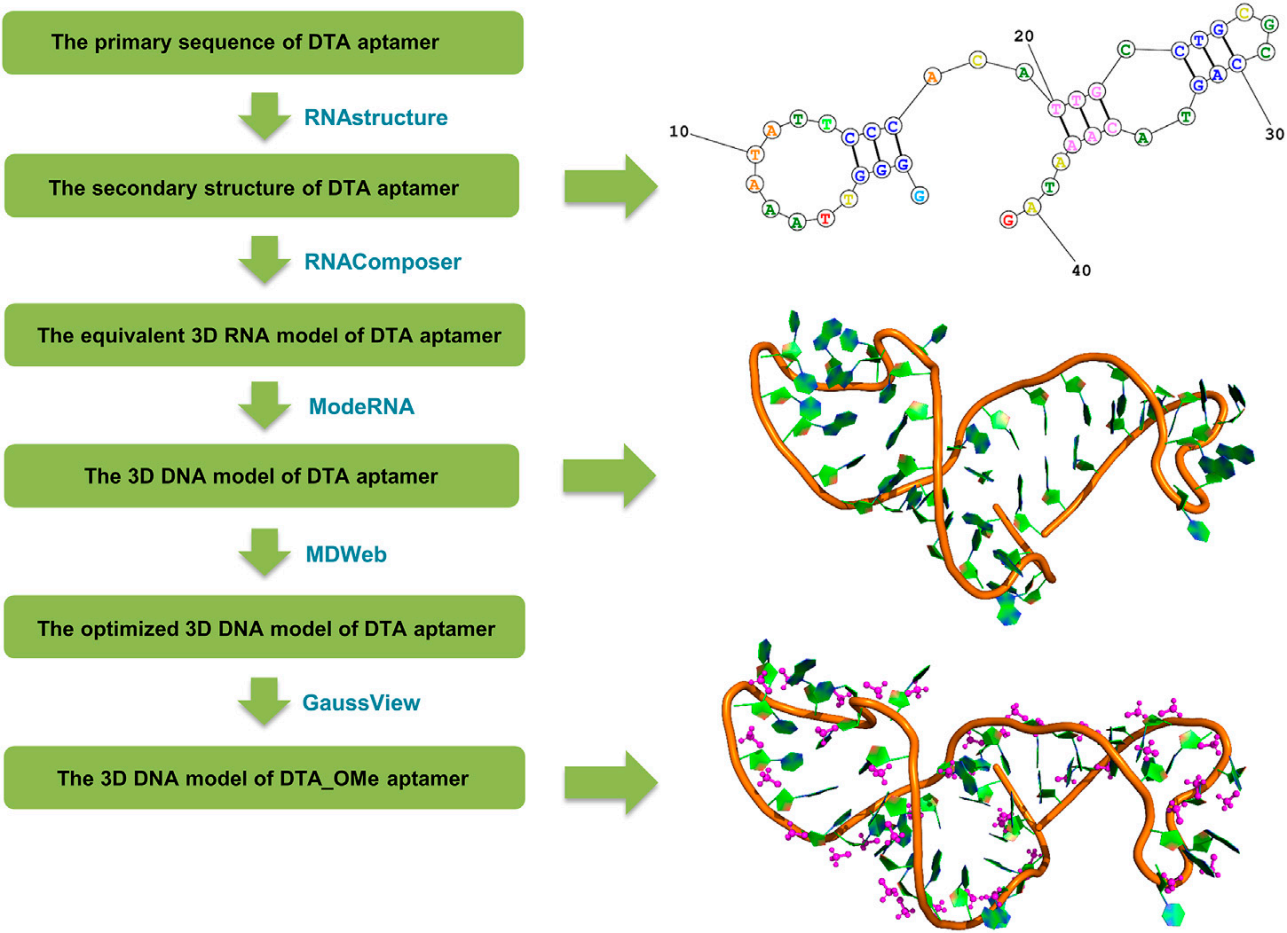
Flow chart of the DTA and DTA_OMe aptamers’ structure prediction originated from the primary sequence.

Based on the primary sequence of the DTA aptamer (5′ GGG GTT AAA TAT TCC CAC ATT GCC TGC GCC AGT ACA AAT AG 3′) (Mor-Vaknin, et al., 2017), the tertiary structures were constructed following a set of steps (Vu, et al., 2018) shown in Figure 2. Firstly, the secondary structure of DTA was generated by the RNAstructure version 6.0.1 webservers (https://rna.urmc.rochester.edu/index.html) (Reuter and Mathews, 2010). Default values were used for all parameters, such as a temperature of 310.15 K, maximum loop size of 30, maximum percent energy difference of 10, maximum number of structure of 20, window size of 3, gamma parameter of 1, and minimum helix length of 3. The structure with the lowest free energy from the three output structures was selected as the secondary structure. Next, based on the secondary structure from the last step, the tertiary RNA structure was constructed using RNAComposer version 1.0 webserver (http://rnacomposer.ibch.poznan.pl/) (Antczak, et al., 2016). Then, the tertiary RNA model was converted to a 3D DNA model using ModeRNA version 1.7.0 webserver (http://iimcb.genesilico.pl/modernaserver) (Rother, et al., 2011). Finally, the 3D DNA model was optimized by running MD simulations using MDWeb version 1.0 webserver (http://mmb.irbbarcelona.org/MDWeb) (Hospital, et al., 2012) that were based on the Ambertools package (version 1.2) (Supplementary Figure S1A). For the modified DTA_OMe model, GaussView version 6.0.1.6 (Dennington et al., 2016) was chosen to modify the 2′ site of DTA deoxyribose by changing hydrogen atoms into -OCH_3_ groups (Supplementary Figure S1B).

### 2.2 Preparation of Force Field Parameters

**FIGURE 3:**
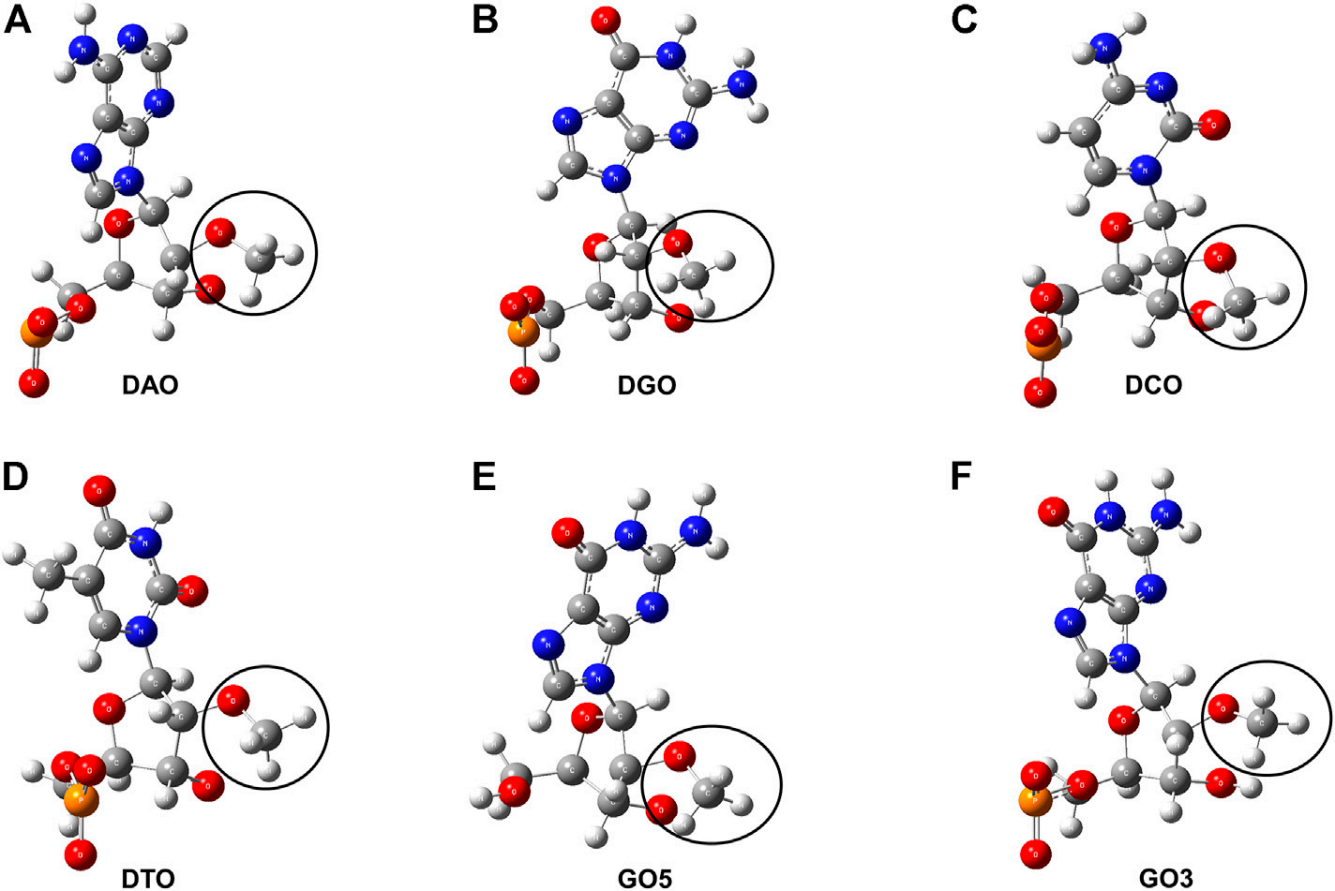
Structure charts of six non-standard nucleotides. **(A)** 2′-O-methyl- deoxyadenosine (DAO). **(B)** 2′-O-methyl-deoxyguanosine (DGO). **(C)** 2′-O-methyl-deoxycytidine (DCO). **(D)** 2′-O-methyl-deoxythymidine (DTO). **(E)** 5′-deoxyguanosine modified with -OCH_3_ group (GO5). **(F)** 3′-deoxyguanosine modified with -OCH_3_ group (GO3). The segments circled by the black curve are all the -OCH_3_ group.

Force field parameters of 2′-OCH_3_-modification nucleotides of DTA_OMe were established to get the integrated MD simulation model, referring to the online AMBER parameter database (http://amber.manchester.ac.uk/) shared by David Case and co-workers (Yildirim, et al., 2014; Zhang, et al., 2014). In our modified model, four deoxyribonucleotides, including deoxyadenosine monophosphate (dAMP), deoxyguanosine monophosphate (dGMP), deoxycytidine monophosphate (dCMP), deoxythymidine monophosphate (dTMP), and two terminal nucleotides, 5′-deoxyguanosine monophosphate (5′-dGMP), 3′-deoxyguanosine monophosphate (3′-dGMP), were modified with 2′-OCH_3_ and a total of six non-standard nucleotides were formed (Figure 3). All these six non-standard nucleotide fragments were optimized by Gaussian16 software package (Frisch et al., 2016), and missing bond parameters were supplemented by the Antechamber and Parmchk2 programs of Amber18 (Case, et al., 2018). To be consistent with the amber99bsc1 force field, partial charges for these six non-standard nucleotides were derived using the RESP approach (Cieplak, et al., 1995) with charges calculated based on a RESP fit to an HF/6-31G* (Hehre and Lathan, 1972; Hariharan and Pople, 1973) electrostatic potential. Specific atom names, atom types, and charges are listed in Supplementary Tables S1, S2 of supporting information.

### 2.3 Protein–DNA Docking

**FIGURE 4:**
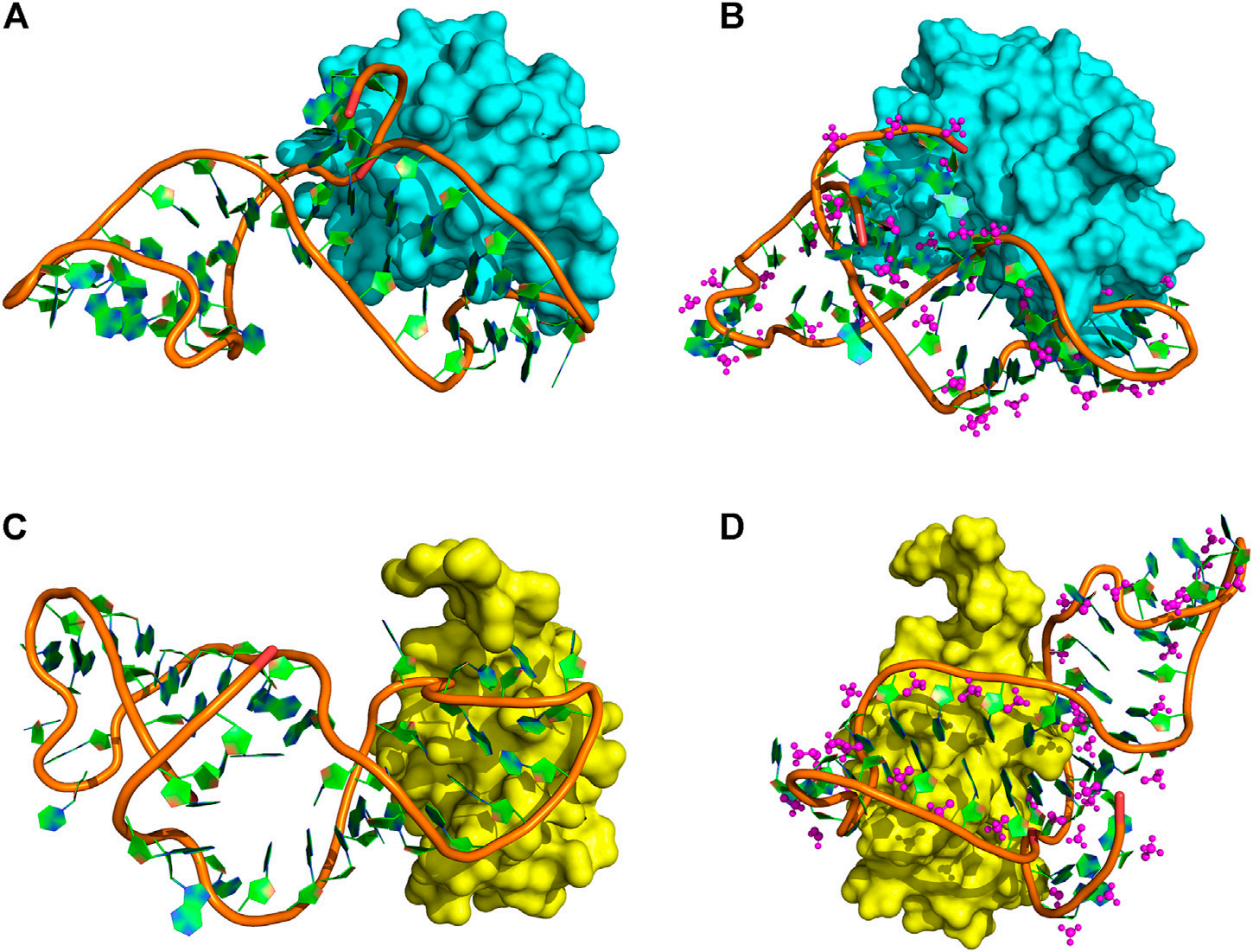
Protein–DNA complex docking models. **(A)** DEK_N/DTA complex. **(B)** DEK_N/DTA_OMe complex. **(C)** DEK_C/DTA complex. **(D)** DEK_C/DTA_OMe complex. Cyan and yellow represent the DEK_N and DEK_C proteins, respectively. The magenta ball-and-stick represents the methoxy groups.

Through testing several popular docking programs including HDOCK (Huang and Zou, 2014; Yan, et al., 2017), HADDOCK, and ZDOCK, the HDOCK server (http://hdock.phys.hust.edu.cn/), developed by Huang Laboratory, was finally chosen for our protein and DNA docking. Especially, HDOCK that has an intrinsic statistical mechanics-based iterative scoring function that can support protein–nucleic docking based on a hybrid docking algorithm of template-based modeling and ab initio free docking (Yan, et al., 2020). Thus, it is more appropriate for our proteins and non-standard DNA docking. Based on this, four protein–DNA interactional models (Figure 4) were set up through molecular docking between DEK_N or DEK_C and DTA or DTA_OMe. Therein, DTA and DTA_OMe are obtained from the aforementioned structure prediction. DEK_N and DEK_C proteins are from the RSCB protein data bank (PDB) (Burley, et al., 2021).

### 2.4 Molecular Dynamics Simulation

All MD simulations were performed using the Amber18 software package using parallel calculations on central- (CPU) and graphics- (GPU) processors (Goetz, et al., 2012; Salomon-Ferrer, et al., 2013). For protein and standard DNA, the amber ff14SB (Maier, et al., 2015) force field and parmbsc1 (Ivani, et al., 2016) nucleic acid parameters were applied. For non-standard or modified DNA, the force field parameters came from the 2′-OCH_3_-modification DNA force field extended by ourselves. The TIP3P water model was applied for waters in a cubic box and the minimum distance between any solute atom and the edge of box was 10 Å or 12 Å (Zhang, et al., 2021) (Supplementary Table S3). Sodium ions were added to neutralize the solvated system and the final ionic concentration was 0.15 M for the solvated system. In order to optimize the structures of aptamers or protein–DNA complexes more adequately, all initial systems were performed to a longer energy minimization process for 1.0 × 10^5^ cycles of the steepest descent (SD) method followed by a further 1.0 × 10^5^ cycles of conjugate gradient algorithms. The energy minimization structures of every aptamer and complex were extracted and the structural changes were investigated. It could be found that DTA and DTA_OMe structures optimized by energy minimization were overlapped with these two aptamer structures obtained by modeling very well (Supplementary Figures S1, S2). And then, based on this, the temperature was slowly and smoothly heated to 300 K using the Langevin dynamic with 5 ps^−1^ collision frequency. During minimization and heating, the whole DNA molecule (Lomzov et al., 2015) was fixed with a constant restraint force of 500 cal/mol/Å^2^. Subsequently, the equilibration was first performed at constant volume, and then at constant pressure (1 bar) by the Berendsen barostat with a pressure relaxation time of 2.0 ps (Dai and Yu, 2020). Finally, the production simulations were carried out at the NPT ensemble. The cutoff value for van der Waals (vdW) and short-range electrostatic interactions was set to 8.0 Å. Long-range electrostatic interactions were treated with the particle-mesh Ewald (PME) summation method. All covalent bonds involving hydrogen atoms were restricted with the SHAKE algorithm. In all simulations, the integral time step was 2 fs, and periodic boundary conditions were used. The simulation time of individual DNA and protein–DNA complexes was 400 and 200 ns (Supplementary Table S3), respectively, and equilibrated trajectories were analyzed by the cpptraj module (Roe and Cheatham, 2013) of Amber18.

### 2.5 MM-PBSA Calculation

At present, the methods widely used to calculate the binding free energy mainly include molecular mechanics poisson–boltzmann surface area (MM-PBSA), free energy perturbation (FEP) (Clark, et al., 2017), thermodynamic integration (TI) (Wu, et al., 2012), and so on. Among these three methods, FEP and TI have a high accuracy while with the slow convergence of the free energy differences and high computational cost computations (Meng, et al., 2011). Considering a good balance between computational efficiency and accuracy, the MM–PBSA method (Wang, et al., 2017) was chosen finally to calculate protein–DNA binding free energy through the MMPBSA. py module (Miller, et al., 2012) of the Amber18 package.

MM–PBSA is a post-processing end-state method and is widely applied as an efficient and reliable free energy simulation method to model molecular recognition, such as protein–DNA binding interaction (Miller, et al., 2012; Hu, et al., 2020; Mousivand, et al., 2022). From the last 50 ns trajectories at an interval of 20 ps, a total of 2,500 snapshots were applied to calculate the binding free energy. The ionic strength was set to 0.15 M, and the external and internal dielectric constants were set to 80 and 1, respectively (Miller, et al., 2012; Yildirim, et al., 2014).

The binding free energy (ΔG_bind_) was computed by the following equation (Chen, et al., 2012), where G_complex_, G_protein_, and G_DNA_ are the free energies of complex, protein, and DNA, respectively.

$${\text{ΔG}}_{\text{bind}}={\text{G}}_{\text{complex}}-{\text{G}}_{\text{protein}}-{\text{G}}_{\text{DNA}}.$$ (1)

In Eq. 1, the corresponding free energy (G_x_ = G_complex_, G_protein_, or G_DNA_) was estimated by MM–PBSA methods:

$${\text{G}}_{\text{X}}=\text{ΔH}–\text{TΔS}\approx {\text{E}}_{\text{MM}}+{\text{G}}_{\text{solv}}–\text{TΔS,}$$ (2)

$${\text{E}}_{\text{MM}}={\text{E}}_{\text{ele}}+{\text{E}}_{\text{vdw}}+{\text{E}}_{\text{int}},$$ (3)

$${\text{G}}_{\text{solv}}={\text{G}}_{\text{PB}}+{\text{G}}_{\text{nonp}}.$$ (4)

Here, T is the temperature, E_MM_ is gas phase molecular mechanical energy, G_solv_ is solvation free energy, and E_ele_, E_vdw_, and E_int_ are the electrostatic energy, van der Waals interaction energy, and internal energy, respectively. In this study, the total value of E_int_ accounting for E_bond_, E_angle_, and E_dihedral_ (bond, angle, and dihedral energies) is zero. The G_solv_ is calculated as the sum of the polar contribution (G_PB_) to the solvation free energy calculated by the Poisson–Boltzmann (PB) model and non-polar contribution (G_nonp_) calculated from a linear relation to the solvent-accessible surface area (SASA):

$${\text{G}}_{\text{nonp}}=\text{γSASA}+\text{β}\text{. }$$ (5)

In Eq. 5, two empirical constants, γ and β, were set as 0.00542 kcal/mol/Å^2^ and 0.92 kcal/mol, respectively. The SASA was determined by a probe radius of 1.4 Å (Chen, et al., 2012; Miller, et al., 2012). The contribution of entropy (-TΔS) toward binding free energy was estimated from normal-mode calculations using the nmode module within Amber18 for the 2,500 snapshots mentioned previously.

## 3 Result and Discussion

### 3.1 Stability Analysis

**FIGURE 5:**
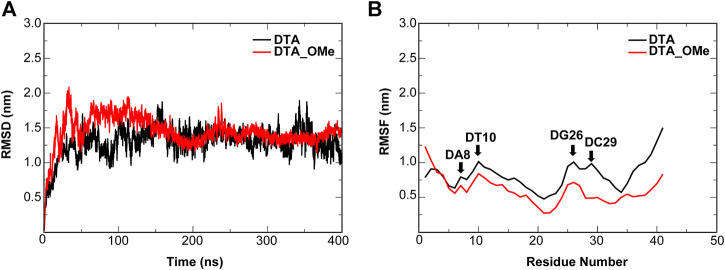
Dynamic trajectory analysis of the DTA and DTA_OMe aptamers. **(A)** RMSD **(B)** RMSF. The nucleotides with the large changes of RMSF before and after modification are labeled.

**FIGURE 6:**
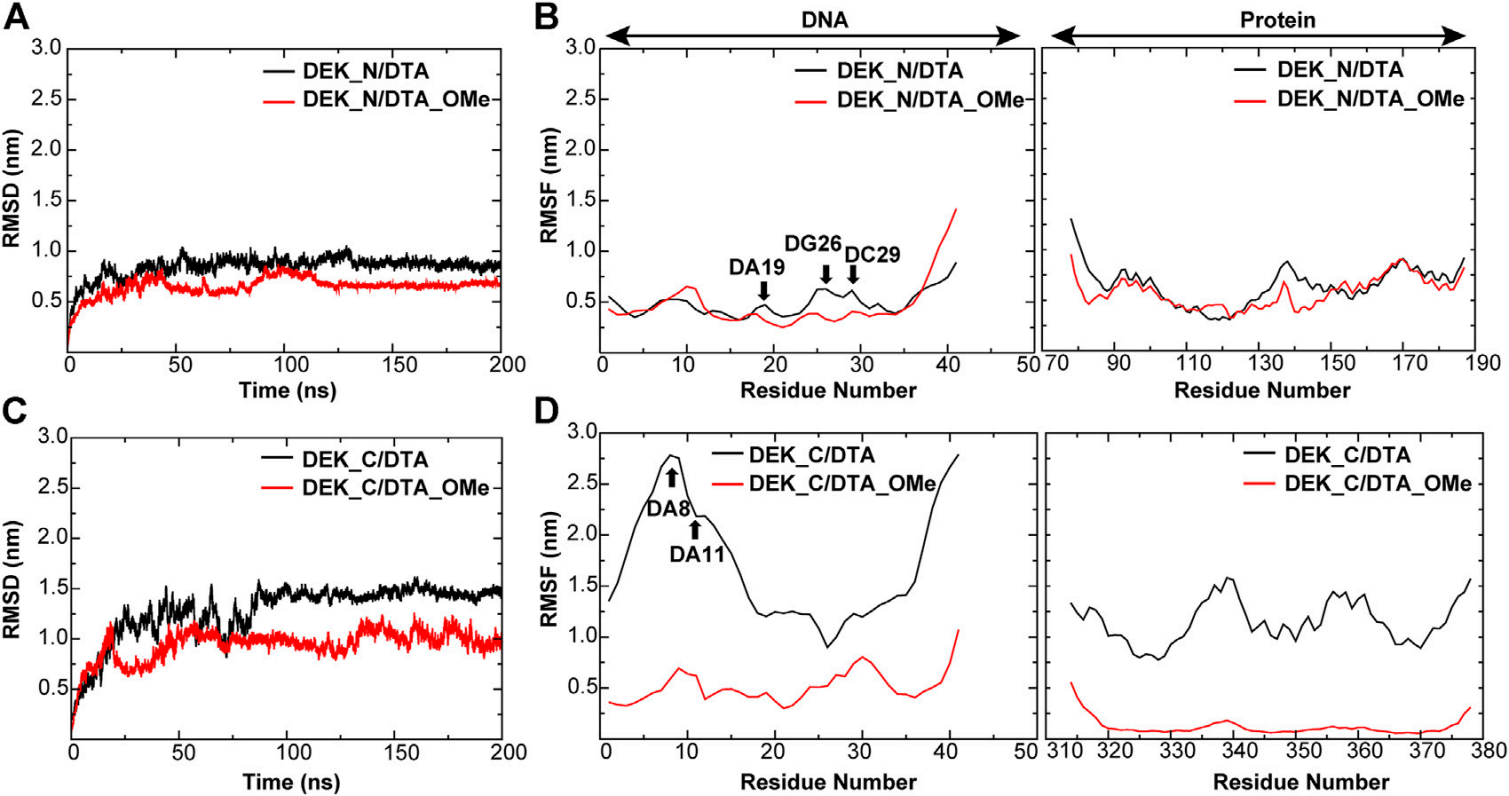
RMSD and RMSF of different protein–DNA complexes. **(A,B)** DEK_N/DTA, DEK_N/DTA_OMe complexes **(C,D)** DEK_C/DTA, DEK_C/DTA_OMe complexes. The nucleotides with the large changes of RMSF before and after modification are labeled.

In order to evaluate the structural stability of aptamers, proteins, and protein–DNA complexes, root mean square deviation (RMSD) and root mean square fluctuation (RMSF) of all heavy atoms were calculated with reference to the minimized structures (Figures 5, 6 and Supplementary Figure S3). The RMSD reflects the stability of the whole protein–-DNA system over time and the RMSF can evaluate the flexibility of a region of protein or DNA.

For individual aptamers, the average RMSD values of DTA and DTA_OMe were 1.35 and 1.34 nm, respectively, but the RMSD curve of DTA_OMe was significantly smoother than that of DTA (Figure 5A). This exhibited that 2′-OCH_3_-modification of DTA would increase the whole stability of this aptamer. As similar as RMSD, RMSF values of modified DTA_OMe were also almost smaller than that of the original DTA in Figure 5B. Particularly, RMSF values of nucleotides DA8, DT10, DG26, and DC29 changed the most before and after modification (Table 1).

**TABLE 1:** RMSF values of some key nucleotides in the individual aptamers and protein–DNA complexes (unit: nm).

Name	DTA	DTA_OMe	Name	DEK_N/DTA	DEK_N/DTA_OMe	Name	DEK_C/DTA	DEK_C/DTA_OMe
DA8	0.76	0.57	DA19	0.47	0.31	DA8	2.78	0.59
DT10	1.01	0.84	DG26	0.63	0.33	DA11	2.18	0.62
DG26	1.01	0.72	DC29	0.61	0.41	—	—	—
DC29	0.98	0.49	—	—	—	—	—	—

As shown in Figure 6 and Table1, for protein–-DNA complexes, the average RMSD values of DEK_N/DTA and DEK_C/DTA were 0.91 and 1.50 nm, respectively. However, after DTA was modified with methoxy groups, the average RMSD values of DEK_N/DTA_OMe and DEK_C/DTA_OMe were reduced to 0.78 and 1.06 nm. Furthermore, RMSF values of the special three nucleotides (DA19, DG26, and DC29) in the DEK_N/DTA_OMe complex were lower than that of the DEK_N/DTA complex, which represented the higher stability. Notably, compared to the DEK_N/DTA and DEK_N/DTA_OMe complexes, the RMSF curve of DEK_C/DTA_OMe was much lower than that of the DEK_C/DTA complex. Also differently, the nucleotides with the largest RMSF value difference switched to DA8 and DA11, which may also contribute most to stability improvement. Therefore, these results indicate that the effect on the stability of DEK_C/DTA_OMe and DEK_C/DTA complexes is greater than DEK_N/DTA and DEK_N/DTA_OMe complexes after 2′-OCH_3_-modification. Interestingly, comparing these four complexes and two individual aptamers, it was found that the stability of DG26, DC29 (N-terminal complexes), and DA8 (C-terminal complexes) that were also included in individual aptamer models (DTA and DTA_OMe) changed greatly before and after modification. From this, we could infer that these three nucleotides may play a significant role in the stability of the DTA aptamer.

### 3.2 Energetic Analysis of Protein–DNA Complexes

#### 3.2.1 Binding Free Energy Analysis

With the MM–PBSA methods, the binding free energies of four complexes (DEK_N/DTA, DEK_N/DTA_OMe, DEK_C/DTA, and DEK_C/DTA_OMe) were computed using the last 50 ns trajectories. The detailed contributions of various energy components computed by the MMPBSA. py program and entropy contributions from the normal-mode analysis are given in Table 2.

**TABLE 2:** Binding free energy (∆G_bind_) and its components (E_ele_, E_vdw_, G_PB_, G_nonp_, E_MM_, G_solv_, and -T∆S) of each protein–DNA complex and the corresponding standard error of the mean (unit: kcal/mol).

Energy	DEK_N/DTA	DEK_N/DTA_OMe	DEK_C/DTA	DEK_C/DTA_OMe
E_ele_	−7,752.67 ± 3.32	−7,356.06 ± 0.77	−3,048.99 ± 0.80	−3,030.96 ± 0.77
E_vdw_	−96.98 ± 0.16	−106.07 ± 0.04	−83.53 ± 0.07	−47.74 ± 0.04
G_PB_	7,706.18 ± 3.27	7,330.67 ± 0.77	3,069.59 ± 0.79	3,023.44 ± 0.75
G_nonp_	−10.82 ± 0.01	−12.63 ± 0.01	−10.15 ± 0.01	−6.99 ± 0.01
E_MM_	−7,849.65 ± 3.35	−7,462.13 ± 0.78	−3,132.52 ± 0.81	−3,078.70 ± 0.77
G_solv_	7,695.36 ± 3.27	7,318.04 ± 0.77	3,059.44 ± 0.79	3,016.45 ± 0.75
∆H	−154.29 ± 0.22	−144.09 ± 0.08	−73.08 ± 0.09	−62.25 ± 0.06
-T∆S	56.57 ± 0.27	53.07 ± 0.40	50.08 ± 0.33	44.89 ± 0.41
∆G_bind_	−97.72	−91.02	−23.00	−17.36

Note: ∆G_bind_ = ∆H - T∆S, ∆H = E_ele_ + E_vdw_ + G_PB_ + G_nonp_, E_MM_ = E_ele_ + E_vdw_, G_solv_ = G_PB_ + G_nonp_.

As shown in Table 2, whether DTA was modified or not, the favorable contributions to binding free energy (∆G_bind_) are the same, mainly coming from the electrostatic energy (E_ele_), the van der Waals interaction energy (E_vdw_), and the non-polar solvation free energy (G_nonp_). Among these three form energies, the electrostatic energy (E_ele_) always had the most negative value, which exactly manifested the strongest driving force to maintain the stability between proteins and aptamers. In contrast with E_ele_, the polar solvation free energy (G_PB_) with the largest positive value made an unfavorable contribution to the binding free energy. In addition, the contribution of entropy changes (-TΔS) to the binding free energy impaired the binding of aptamers and proteins.

For N-terminal, the binding free energy of DEK_N/DTA (−97.72 kcal/mol) was more negative than that of DEK_N/DTA_OMe (−91.02 kcal/mol) and the difference value between them was only −6.70 kcal/mol. Similarly, for C-terminal, the binding free energy of DEK_C/DTA (−23.00 kcal/mol) was also more negative than that of DEK_C/DTA_OMe (−17.36 kcal/mol) and the difference value between them was smaller, only −5.64 kcal/mol. These results indicate that the DEK_N protein has a lower binding free energy and higher affinity to DTA or DTA_OMe than DEK_C. However, the binding ability of DEK_N or DEK_C to DTA_OMe was slightly decreased due to the 2′-OCH_3_-modification, but this effect was rather limited. In general, after the DTA aptamer was modified by methoxy groups, the affinity of DTA_OMe with DEK_N and DEK_C proteins could still be maintained at a considerable level, and their stability was greatly enhanced.

#### 3.2.2 Per-Residue Free Energy Decomposition

**FIGURE 7:**
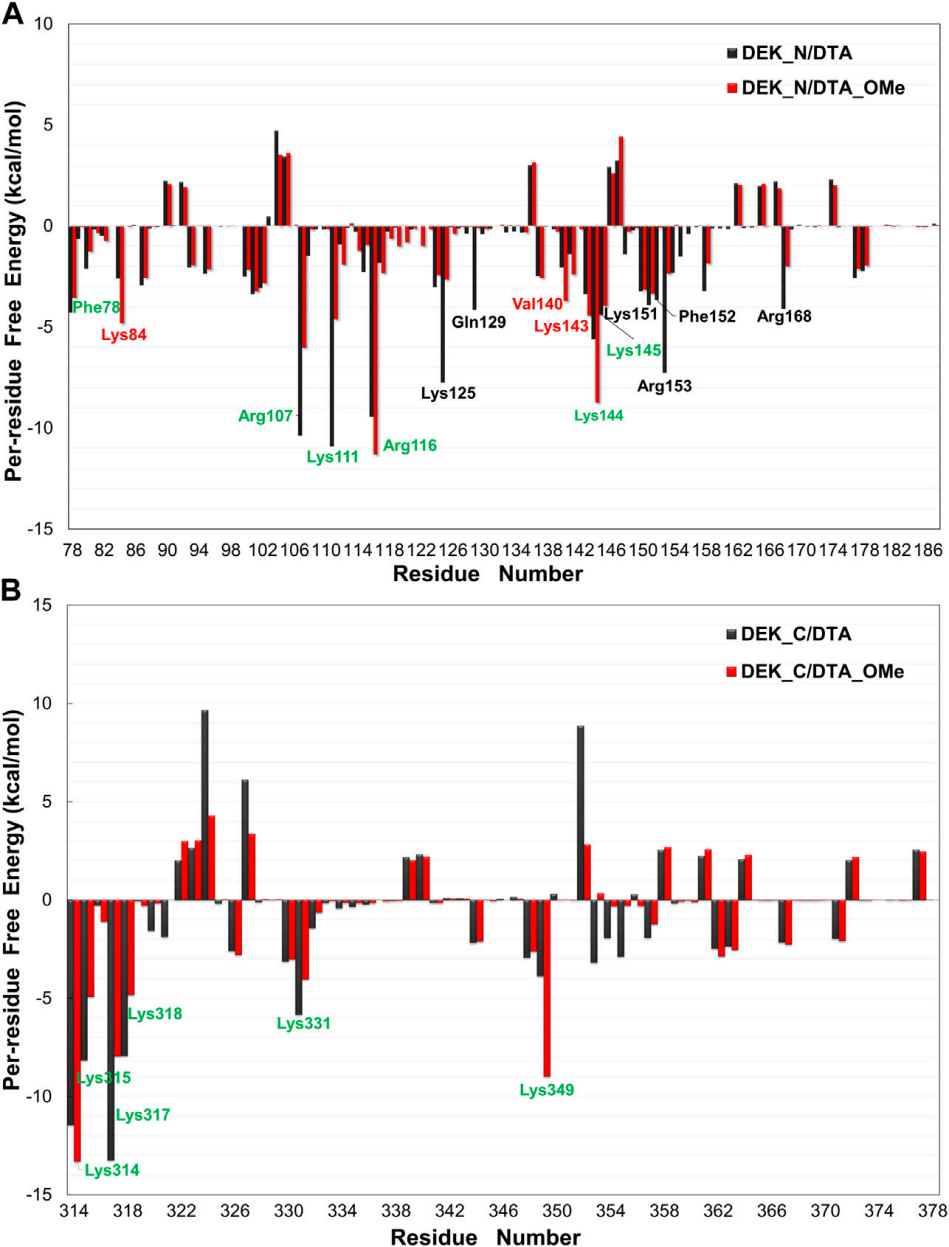
Per-residue free energy decomposition diagrams of different protein–DNA complexes. **(A)** DEK_N/DTA, DEK_N/DTA_OMe complexes. **(B)** DEK_C/DTA, DEK_C/DTA_OMe complexes. The residues with energies less than −3.50 kcal/mol are marked with different colors. Among them, the black and red letters represent the different main residues present in the DEK_N/DTA and DEK_N/DTA_OMe complexes, respectively. The green letters represent the main residues present in both complexes.

In order to explore further binding mechanisms between DEK_N or DEK_C proteins and DTA or DTA_OMe aptamers and to identify key residues of these protein–DNA interaction interfaces, the contribution of each individual residue toward binding energies was further analyzed in detail (Figure 7 and Supplementary Tables S4, S5). The values of E_MM_ and G_solv_ were decomposed on each residue basis into contributions from the internal energy, van der Waals energy, electrostatic energy, polar solvation free energy, and non-polar solvation free energy. In Figure 7, amino acid residues making a major contribution toward binding energies with energies less than -3.50 kcal/mol are highlighted with different colors.

As shown in Figure 7A, for N-terminal, a total of twelve amino acid residues (Phe78, Arg107, Lys111, Arg116, Lys125, Gln129, Lys144, Lys145, Lys151, Phe152, Arg153, and Arg168) made a major contribution to the binding energy with less than −3.50 kcal/mol in the DEK_N/DTA complex. In the same way, a total of nine amino acid residues (Phe78, Lys84, Arg107, Lys111, Arg116, Val140, Lys143, Lys144, and Lys145) made a major contribution to the binding energy in the DEK_N/DTA_OMe complex. For C-terminal, in both DEK_C/DTA and DEK_C/DTA_OMe complexes, the major contribution toward binding energies came from six amino acid residues (Lys314, Lys315, Lys317, Lys318, Lys331, and Lys349), which were located in the C-terminal DNA-binding region (270-350) of the DEK protein predicted in the experiments (Waldmann, et al., 2004).

Interestingly, these residues are all lysine with positive charges, which can be attracted more easily by the phosphate backbone of DNA with an opposite negative charge. This analysis suggested that these residues mentioned previously helped to maintain the stability and affinity between DEK_N or DEK_C proteins and DTA or DTA_OMe aptamers. This conclusion was also verified in the following hydrogen bond analysis section. Moreover, it can be seen from Figure 7 that the key amino acids in DEK_N/DTA and DEK_N/DTA_OMe complexes are significantly more than DEK_C/DTA and DEK_C/DTA_OMe. This indicates that DTA or DTA_OMe has a higher affinity for DEK_N proteins (i.e. lower binding free energies).

### 3.3 Protein–DNA Complexes Hydrogen Bond Analysis

As like a “bridge”, the hydrogen bond is extremely considerable when the interactions between proteins and DNA are analyzed. Generally, these hydrogen bonds include both non-specific interactions mainly between proteins and the DNA sugar/phosphate backbone and specific interactions mainly between proteins and the DNA base. Herein, we analyzed the hydrogen bond interactions between DEK_N or DEK_C proteins and DTA or DTA_OMe aptamers, and listed the detailed information in Supplementary Tables S6–S9, in which the hydrogen bond occupancy exceeded 10% during the last 50 ns trajectories of all the simulations.

**FIGURE 8:**
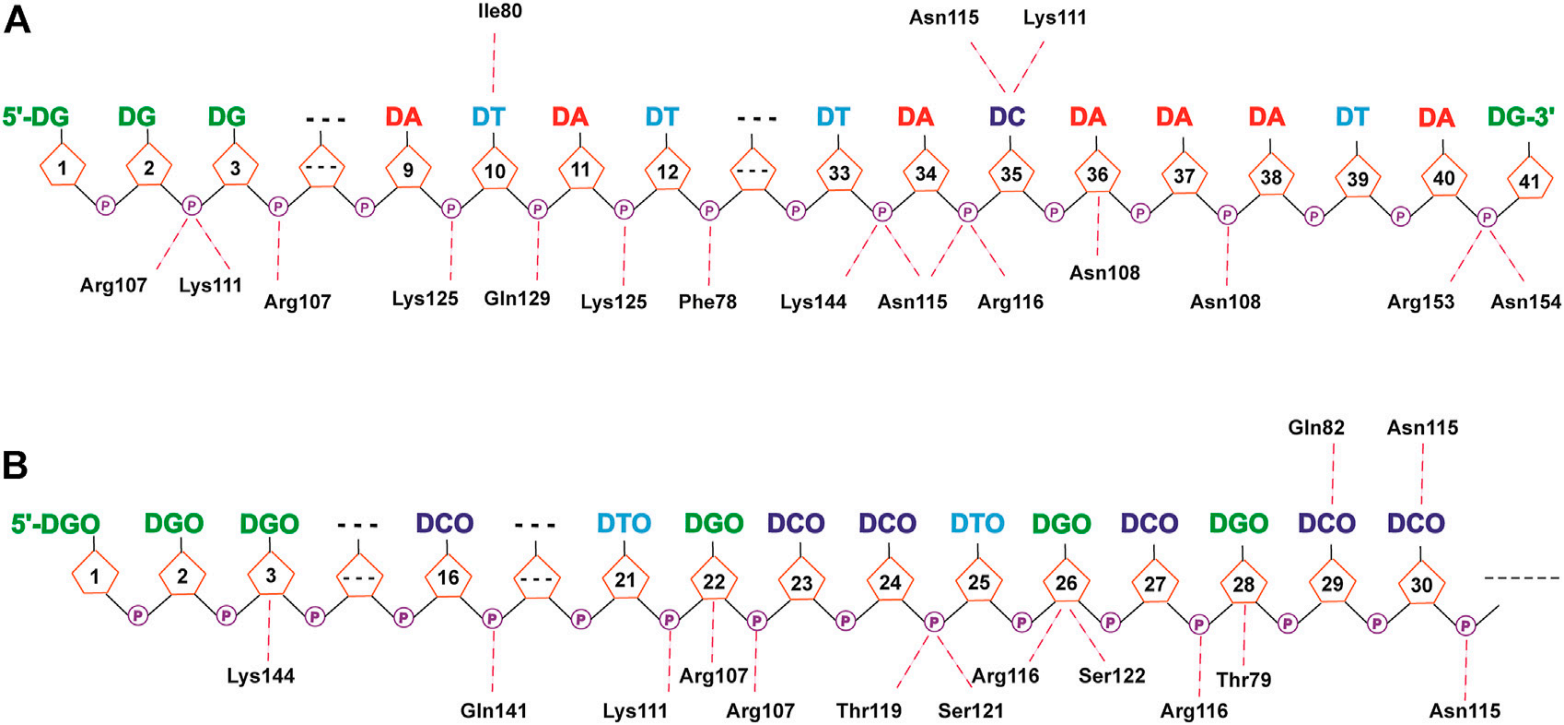
Hydrogen bond networks for protein–DNA complexes. **(A)** DEK_N/DTA. **(B)** DEK_N/DTA_OMe. Specific hydrogen bonds are listed above the DNA strand, and non-specific hydrogen bonds are listed below the DNA strand.

**FIGURE 9:**
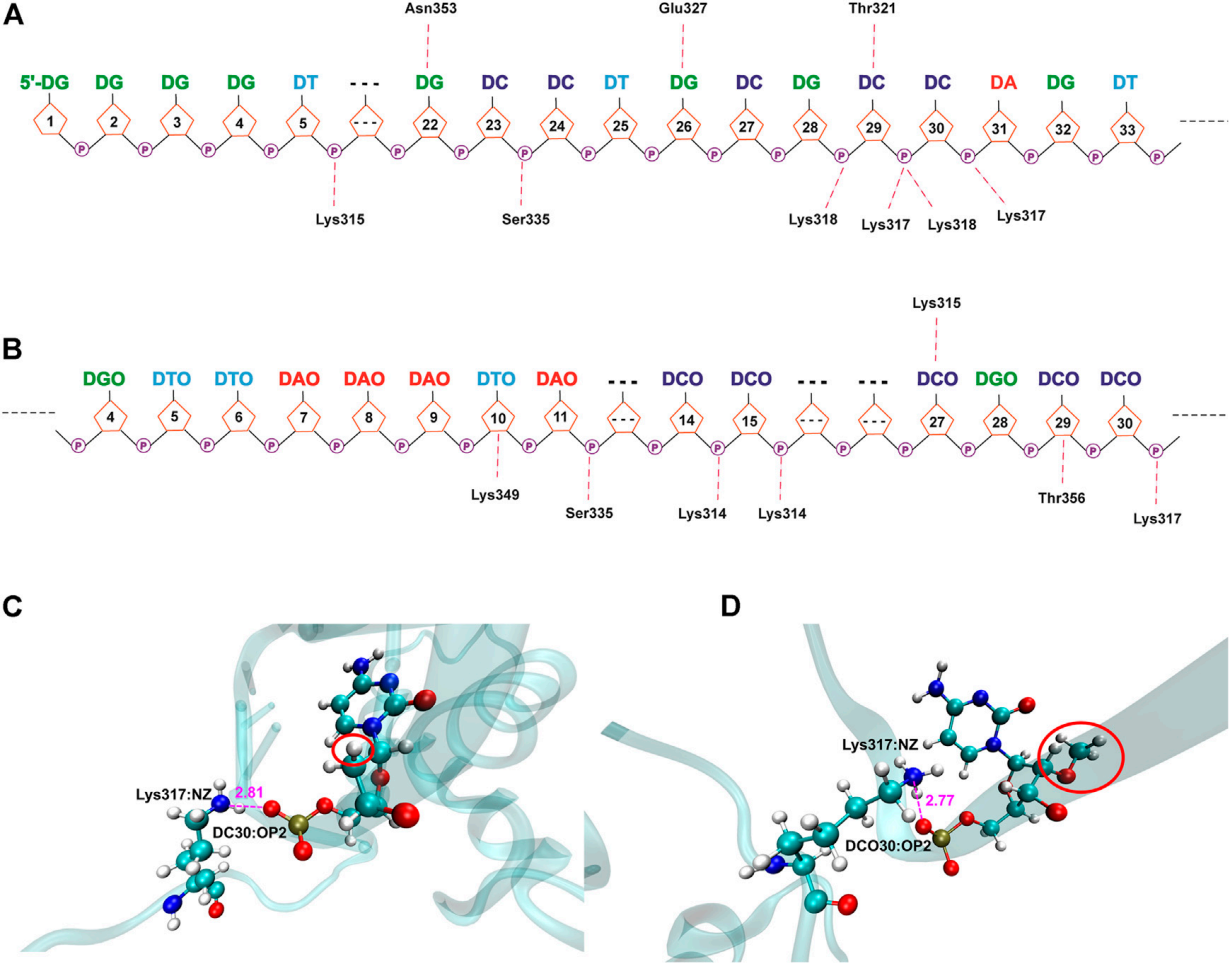
Hydrogen bond networks for protein–DNA complexes. **(A)** DEK_C/DTA. **(B)** DEK_C/DTA_OMe. Specific hydrogen bonds are listed above the DNA strand, and non-specific hydrogen bonds are listed below the DNA strand. **(C,D)** The detailed hydrogen bond interaction diagram between DCO30 and Lys317 in DEK_C/DTA and DEK_C/DTA_OMe complexes.

As shown in Figures 8, 9 and Supplementary Tables S6–S9, due to the electronegativity of the sugar/phosphate backbone of DNA, more non-specific hydrogen bonds existed than specific hydrogen bonds in all four protein–DNA complexes. For N-terminal complexes, DEK_N/DTA and DEK_N/DTA_OMe had 26 and 19 non-specific hydrogen bonds, respectively. The hydrogen bonds with the highest occupancy were DA40-Arg153 (63.67%) and DCO27-Arg116 (87.66%) in these two complexes. For C-terminal complexes, there were 12 non-specific hydrogen bonds in DEK_C/DTA complex, and among them, DC29-Lys318 had the highest occupancy (59.27%). In the DEK_C/DTA_OMe complex, there were 14 non-specific hydrogen bonds, of which the hydrogen bond with the highest occupancy was DTO10-Lys349 (27.59%).

Moreover, a common hydrogen bond was formed in the DEK_C/DTA and DEK_C/DTA_OMe complexes, namely DC30-Lys317 (Figure 9). For this hydrogen bond, in the DEK_C/DTA, the distance between the oxygen atom (OP2) of DC30 and the nitrogen atom (NZ) of Lys317 was 2.81 Å, and the hydrogen bond occupancy rate was 39.04%. However, the distance was shortened to 2.77 Å and the occupancy increased to 42.02% in DEK_C/DTA_OMe. Structurally, as shown in Figure 9, it could be seen that the steric hindrance was increased after DC30 modified by the methoxy group, which led to the twisting of the pentabasic sugar ring structure. This structural change drove cytosine and phosphate to rotate toward the opposite side of the methoxy group. Therefore, the distance between OP2 and NZ was shortened, and simultaneously, the stability of DC30-Lys317 was improved in the DEK_C/DTA_OMe complex.

In a word, most of these non-specific hydrogen bonds were generated by positively-charged residues (e.g., Lys and Arg) in DEK and negatively-charged phosphate backbone atoms of DTA and DTA_OMe. They played an important role in maintaining the stability between the DEK protein and the two aptamers. Furthermore, the relation between positively-charged residues of the DEK protein and negatively-charged phosphate groups of DTA and DTA_OMe aptamers may have contributed to the formation of electrostatic interactions (Cherstvy, 2009).

Except the non-specific hydrogen bonds discussed previously, the specific interactions are important for molecular recognition processes in protein–DNA complexes, which affect the specificity and affinity of DNA to proteins. For N-terminal complexes, there were six specific hydrogen bonds in DEK_N/DTA, namely Ile80-DT10@H3, DC35-Lys111@HZ3, DC35-Lys111@HZ1, DC35-Lys111@HZ2, Asn115-DC35, and DT10-Ile80@H (Supplementary Table S6). However, the DEK_N/DTA_OMe complex contained only two (Gln82-DCO29 and Asn115-DCO30) specific hydrogen bonds (Supplementary Table S7). For C-terminal complexes, DEK_C/DTA possessed Thr321-DC29, Asn353-DG22@H21, Asn353-DG22@H1, Glu327-DG26@H1, and Glu327-DG26@H21, with a total of five specific hydrogen bonds (Supplementary Table S8). The DEK_C/DTA_OMe covered one specific hydrogen bond, namely DCO27-Lys315 (Supplementary Table S9). After DTA was modified with methoxy groups, the number of specific hydrogen bonds with DEK_N and DEK_C was significantly reduced. Furthermore, as shown in Table 2, DTA_OMe had higher binding free energies to DEK_N and DEK_C proteins compared to DTA, and correspondingly, the affinities of DTA_OMe to these two proteins were also reduced. Therefore, in order to obtain the aptamers with higher affinities, additional mutations were carried out for all DNA bases of specific hydrogen bonds based on the protein–DNA interaction mechanisms in the following aptamer design section.

### 3.4 Mutational Study

In order to enhance the affinity of DTA_OMe or DTA to the DEK protein and provide some theoretical basis for the design of new aptamers, we carried out a single site mutation on the DTA_OMe and DTA aptamers. According to the hydrogen bond results of the four complexes aforementioned, eight DNA bases forming specific hydrogen bonds on DTA_OMe or DTA as mutation sites were selected. Then, on the basis of the structure of the corresponding wild type complexes, each of these eight bases was randomly mutated into other three bases, and a total of 24 mutants were obtained (Supplementary Table S10). The similarly classical MD simulations and MM–PBSA calculations were performed to help reveal what role these key DNA bases exactly played in the interaction mechanisms.

**FIGURE 10:**
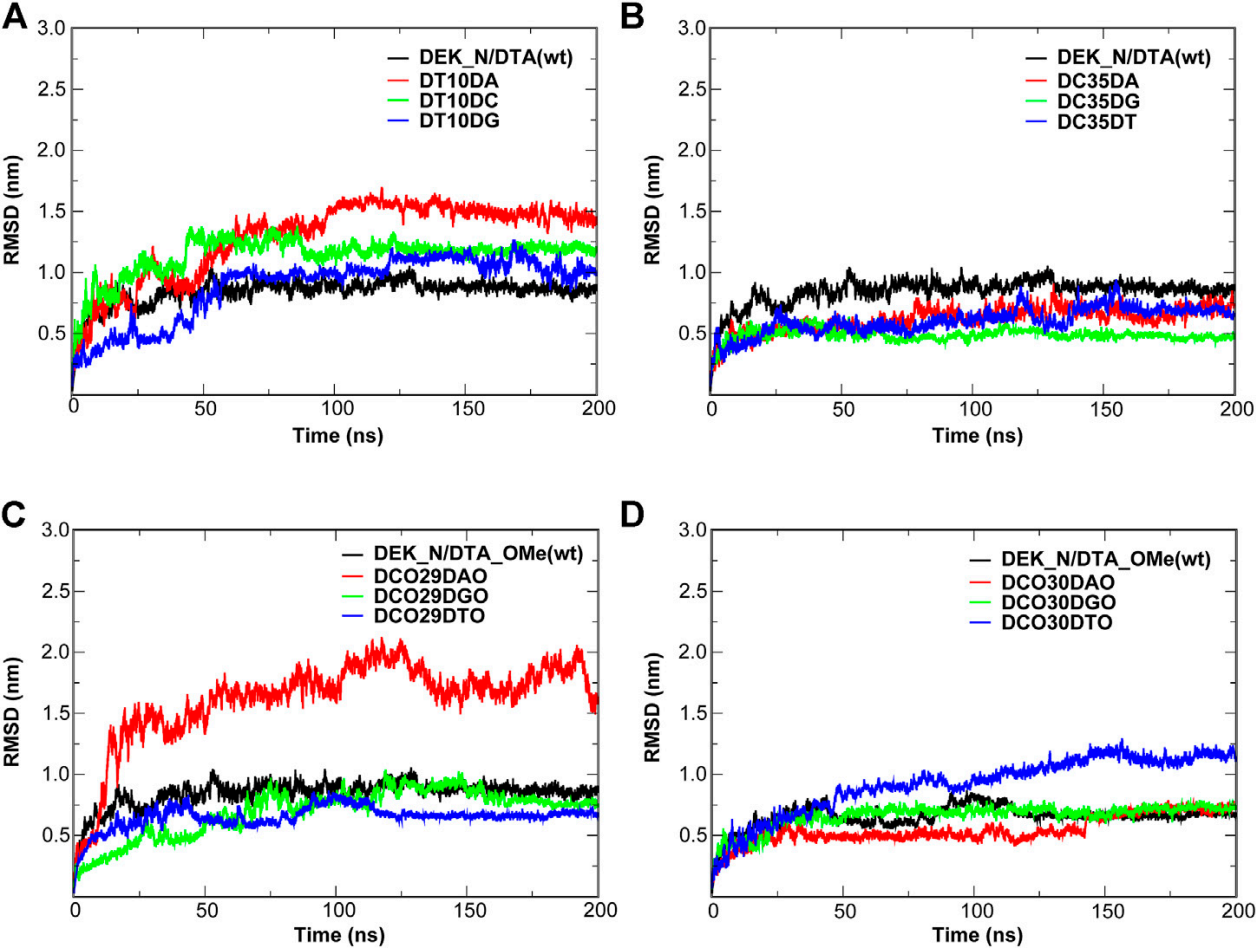
RMSD of the wild type complexes and the corresponding mutants. **(A,B)** DEK_N/DTA complex and its mutants. **(C,D)** DEK_N/DTA_OMe complex and it mutants.

**FIGURE 11:**
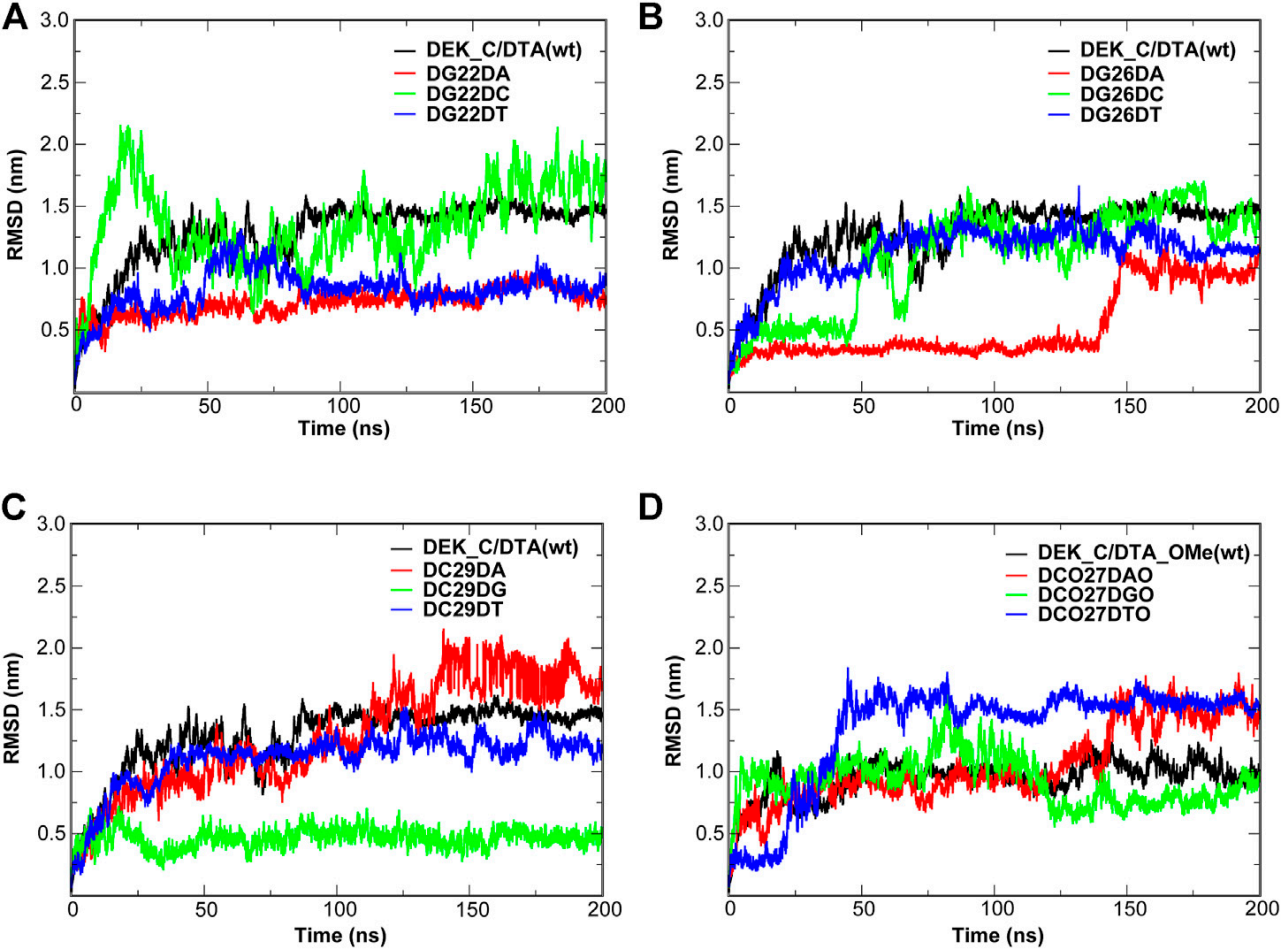
RMSD of the wild type complexes and the corresponding mutants. **(A–C)** DEK_C/DTA complex and its mutants. **(D)** DEK_C/DTA_OMe complex and its mutants.

Figures 10, 11 give out the RMSD curve fluctuations for all mutants and the corresponding wild type complexes. Compared to the wild type complexes, the majority of mutants represented a comparative or better stability. Among such mutations, it was found that besides the 10th mutation site of N-terminal, at least one type of mutant on the other each mutation site would increase the stability of the interaction systems compared to wild type complexes. Especially, for N-terminal, these mutants were DC35DG, DCO29DTO, and DCO30DAO (Figure 10). Likewise, for C-terminal, these mutants were DG22DA, DG26DA, DC29DG, and DCO27DGO (Figure 11).

In order to further evaluate the affinity of different mutants for DEK_N or DEK_C, we selected seventeen mutants where their RMSD were relatively flat or smaller than wild types to calculate their binding energies (∆G) using the MM–PBSA method. The energy difference (∆∆G) between each mutant and corresponding wild type complex is also calculated and listed in Table 3 as well. However, considering the calculation of entropy contribution was extremely time-consuming and computationally expensive, we ignored it here. As can be seen from Table 3, except for DG22DA, the binding free energies of other mutants were all negative, and the mutant with the lowest energy was DCO29DTO (−187.26 kcal/mol). However, there were only 7 mutants with negative ∆∆G values, namely DC35DT, DCO29DGO, DCO29DTO, DCO30DAO, DCO30DGO, DCO27DGO, and DCO27DTO, but the mutant with the smallest ∆∆G was also DCO29DTO (−43.17 kcal/mol). It was not difficult to see that these seven mutants were derived from modified DTA_OMe except for DC35DT. This showed that the –OCH_3_ group had a great influence on mutants, which could greatly improve their thermal stability or affinity.

**TABLE 3:** Energy distribution of the wild type complexes and the corresponding mutants. ∆∆G means the energy difference between each mutant and its corresponding wild type (unit: kcal/mol).

Complex	Mutation Site	Mutant	E_ele_	E_vdw_	G_PB_	G_nonp_	∆G	∆∆G
DEK_N/DTA (wt)	—	—	−7,752.67	−96.98	7,706.18	−10.82	−154.29	—
DEK_N/DTA	DT10	DT10DA	−6,472.18	−56.85	6,428.78	−7.74	−107.99	46.30
		DT10DC	−5,942.84	−105.98	5,928.36	−11.54	−132.00	22.29
		DT10DG	—	—	—	—	—	—
	DC35	DC35DA	−7,229.86	−78.03	7,185.82	−9.36	−131.43	22.86
		DC35DG	−7,212.93	−116.12	7,197.80	−11.87	−143.12	11.17
		DC35DT	−7,696.46	−115.38	7,654.10	−12.46	−170.20	−15.91
DEK_N/DTA_OMe (wt)	—	—	−7,356.06	−106.07	7,330.67	−12.63	−144.09	—
DEK_N/DTA_OMe	DCO29	DCO29DAO	—	—	—	—	—	—
		DCO29DGO	−8,057.47	−132.02	8,043.25	−16.34	−162.58	−18.49
		DCO29DTO	−8,287.57	−121.26	8,237.71	−16.14	−187.26	−43.17
	DCO30	DCO30DAO	−7,809.05	−107.67	7,756.59	−13.08	−173.21	−29.12
		DCO30DGO	−8,456.74	−133.51	8,440.55	−17.01	−166.71	−22.62
		DCO30DTO	—	—	—	—	—	—
DEK_C/DTA (wt)	—	—	−3,048.99	−83.53	3,069.59	−10.15	−73.08	—
DEK_C/DTA	DG22	DG22DA	−1,668.19	−72.00	1755.50	−9.37	5.94	79.02
		DG22DC	—	—	—	—	—	—
		DG22DT	−3,446.97	−46.91	3,432.10	−6.68	−68.46	4.62
	DG26	DG26DA	−3,196.70	−44.45	3,182.18	−6.82	−65.79	7.29
		DG26DC	—	—	—	—	—	—
		DG26DT	−3,592.66	−42.40	3,573.68	−6.66	−68.04	5.04
	DC29	DC29DA	—	—	—	—	—	—
		DC29DG	−2,568.63	−64.55	2,604.98	−9.38	−37.58	35.50
		DC29DT	−2,329.24	−39.89	2,313.82	−5.34	−60.65	12.43
DEK_C/DTA_OMe (wt)	—	—	−3,030.96	−47.74	3,023.44	−6.99	−62.25	—
DEK_C/DTA_OMe	DCO27	DCO27DAO	—	—	—	—	—	—
		DCO27DGO	−2,361.78	−43.68	2,347.16	−6.72	−65.02	−2.77
		DCO27DTO	−2,919.51	−69.77	2,932.76	−9.79	−66.31	−4.06

Note: ∆G = E_ele_ + E_vdw_ + G_PB_ + G_nonp_, ∆∆G = ∆G_mut_ - ∆G_wt_.

Considering RMSD and ∆∆G comprehensively, the DCO29DTO mutant with high affinity and stability is recommended. Consistently, RMSF values of DC29 in both the DTA_OMe aptamer and the DEK_N/DTA_OMe complex were reduced greatly after being modified by the –OCH_3_ group as mentioned previously (Figure 5). Such consistency demonstrated that the 29th nucleotide on DTA is a promising mutation site, especially after –OCH_3_ modification. Additionally, DCO29DTO in which the 29th base is replaced from cytosine to thymine of DTA_OMe is an ideal mutant with higher stability and affinity to the DEK_N protein.

## 4 Conclusion

Considering the advantages of easy synthesis and modification, the DTA aptamer has attracted more and more attention as a potential drug candidate for the treatment of inflammatory arthritis. However, the poor stability of DTA *in vivo* has greatly limited its clinical application. In order to discover new aptamers with better stability and affinity, we got insights into the exact DEK–DTA interaction mechanisms through MD simulations, and put forward a more promisingly mechanism-based and structure-based aptamer design strategy. In particular, our research shows that the 2′-OCH_3_-modified DTA can definitely enhance its stability on the premise of comparative affinity. The electrostatic interactions and non-specific hydrogen bonds derived primarily from positively-charged residues of DEK and negatively-charged phosphate backbone of aptamers cooperate to maintain the stability of DEK–-DTA. Due to its outstanding affinity and stability to the DEK_N protein, DCO29DTO is selected out from 24 mutants which were designed from 8 bases participating in specific hydrogen bonds.

By experimental methods, it is difficult to determine key residues in the DEK–DTA interaction. Therefore, this study provides effective theoretical guidance for the exploration of the DEK–DTA binding mechanism, and offers a more efficient design strategy of new aptamers with higher anti-inflammatory potential combined traditional SELEX technology with MD simulations. Furthermore, the extended 2′-OCH_3_-DNA force field parameters can be applied universally by other researchers. In the future, it is also believed that the double-site or multi-site mutations on DTA or DTA_OMe aptamers will be planned to carry out, which may provide more valuable information for us to explore the other potential roles of the DTA aptamer.

## Data Availability

The original contributions presented in the study are included in the article/Supplementary Material; further inquiries can be directed to the corresponding authors.
