# Supplementary material for: Protein DEK and DTA Aptamers: Insight Into the Interaction Mechanisms and the Computational Aptamer Design
Source: Front Mol Biosci. 2022 Jul 19;9:946480. doi: 10.3389/fmolb.2022.946480 (PMC9345330; doi:10.3389/fmolb.2022.946480)
Supplement: Supplementary file 1 [file DataSheet1.pdf]

Supplementary data

Table of Contents

Supplementary Figure:

Supplementary Figure S1. Schematic diagram of aptamer structures. **(A, B)** DTA and DTA_OMe aptamer structures obtained by modeling. **(C, D)** DTA and DTA_OMe aptamer structures obtained by energy minimization. **(E)** DTA and **(F)** DTA_OMe aptamer structures alignment by modeling and minimization optimization.

Supplementary Figure S2. Structure diagram of all protein-DNA complex models after energy minimization. **(A)** DEK_N/DTA, **(B)** DEK_N/DTA_OMe, **(C)** DEK_C/DTA, **(D)** DEK_C/DTA_OMe. Cyan and yellow cartoons represent DEK_N and DEK_C proteins respectively.

Supplementary Figure S3. RMSD curves of different proteins. **(A)** DEK_N, **(B)** DEK_C.

Supplementary Tables:

Supplementary Table S1. Detail parameters of 2’-OCH_3 modified nonstandard nucleotides (DAO, DGO and DCO) in the established force field of DTA_OMe.

Supplementary Table S2. Detail parameters of 2’-OCH_3 modified nonstandard nucleotides (DTO, GO5 and GO3) in the established force field of DTA_OMe.

Supplementary Table S3. Simulation details of protein, DNA aptamer and protein-DNA complex models in solvent.

Supplementary Table S4. Per-residue free energy decomposition of DEK_N/DTA and DEK_N/DTA_OMe complexes (only show the negative energy).

Supplementary Table S5. Per-residue free energy decomposition of DEK_C/DTA and DEK_C/DTA_OMe complexes (only show the negative energy).

Supplementary Table S6. Detailed information of hydrogen bonds in DEK_N/DTA complex.

Supplementary Table S7. Detailed information of hydrogen bonds in DEK_N/DTA_OMe complex.

Supplementary Table S8. Detailed information of hydrogen bonds in DEK_C/DTA complex.

Supplementary Table S9. Detailed information of hydrogen bonds in DEK_C/DTA_OMe complex.

Supplementary Table S10. Sequences and modification schemes of mutants (the cyan boxes are mutation sites and red letters are nucleotides modified with 2′-OCH_3 in the deoxyribose sugar unit).

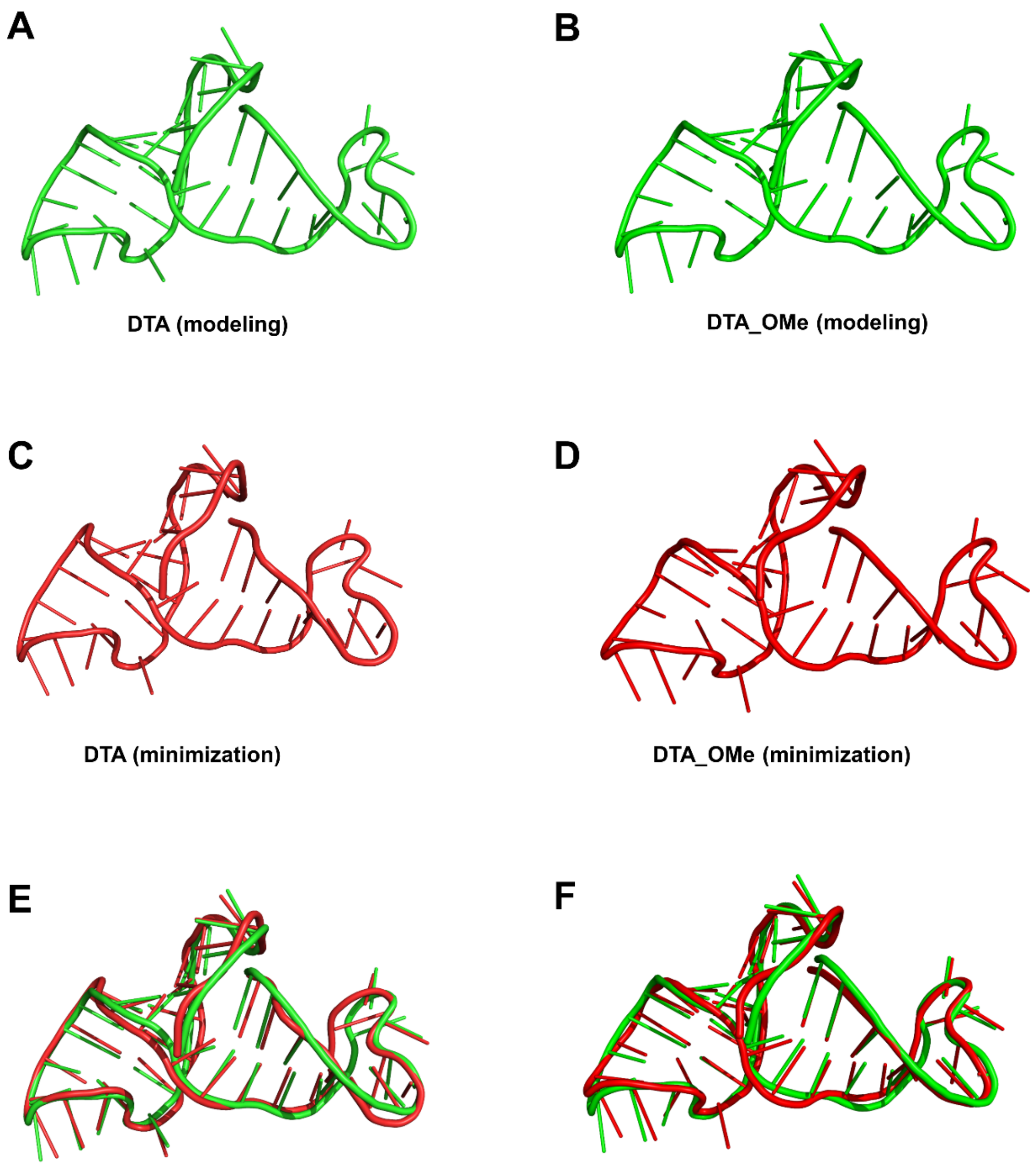

Supplementary Figure S1. Schematic diagram of aptamer structures. **(A, B)** DTA and DTA_OMe aptamer structures obtained by modeling. **(C, D)** DTA and DTA_OMe aptamer structures obtained by energy minimization. **(E)** DTA and **(F)** DTA_OMe aptamer structures alignment by modeling and minimization optimization.

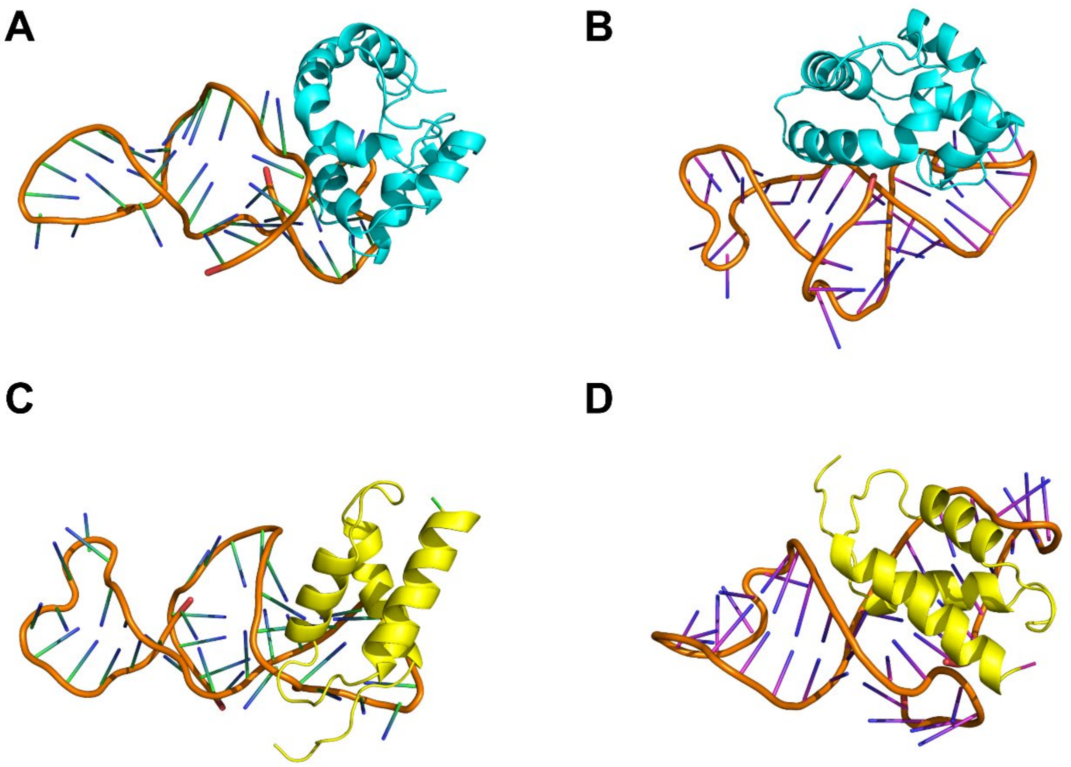

Supplementary Figure S2. Structure diagram of all protein-DNA complex models after energy minimization. **(A)** DEK_N/DTA, **(B)** DEK_N/DTA_OMe, **(C)** DEK_C/DTA, **(D)** DEK_C/DTA_OMe. Cyan and yellow cartoons represent DEK_N and DEK_C proteins respectively

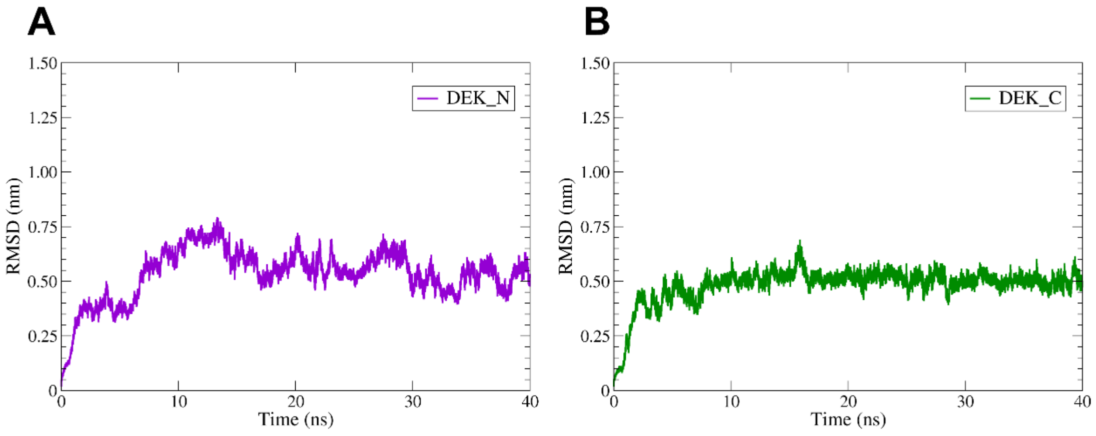

Supplementary Figure S3. RMSD curves of different proteins. **(A)** DEK_N, **(B)** DEK_C.

Supplementary Table S1. Detail parameters of 2'-OCH3 modified nonstandard nucleotides (DAO, DGO and DCO) in the established force field of DTA_OMe.

Serial number	DAO Atom name	Atom type	Atom charge	DGO Atom name	Atom type	Atom charge	DCO Atom name	Atom type	Atom charge
1	OP1	O2	-0.776100	OP1	O2	-0.776100	OP1	O2	-0.776100
2	P	P	1.165900	P	P	1.165900	P	P	1.165900
3	OP2	O2	-0.776100	OP2	O2	-0.776100	OP2	O2	-0.776100
4	O5'	OS	-0.495400	O5'	OS	-0.495400	O5'	OS	-0.495400
5	C5'	CI	-0.006900	C5'	CI	-0.006900	C5'	CI	-0.006900
6	H5'	H1	0.075400	H5'	H1	0.075400	H5'	H1	0.075400
7	H5"	H1	0.075400	H5"	H1	0.075400	H5"	H1	0.075400
8	C4'	CT	0.162900	C4'	CT	0.162900	C4'	CT	0.162900
9	H4'	H1	0.117600	H4'	H1	0.117600	H4'	H1	0.117600
10	C3'	CE	0.071300	C3'	CE	0.071300	C3'	CE	0.071300
11	H3'	H1	0.098500	H3'	H1	0.098500	H3'	H1	0.098500
12	C2'	CT	1.004252	C2'	CT	1.047946	C2'	CT	-0.337646
13	H2'	H1	0.454992	H2'	H1	0.490953	H2'	H1	0.733344
14	O2'	OS	-1.265496	O2'	OS	-1.275964	O2'	OS	0.581614
15	CM2	CT	0.807210	CM2	CT	0.809411	CM2	CT	-0.797516
16	H11	H1	-0.172799	H11	H1	-0.175796	H11	H1	0.001395
17	H12	H1	-0.172799	H12	H1	-0.175796	H12	H1	0.001395
18	H13	H1	-0.172799	H13	H1	-0.175796	H13	H1	0.001395
19	O3'	OS	-0.523200	O3'	OS	-0.523200	O3'	OS	-0.523200
20	O4'	OS	-0.369100	O4'	OS	-0.369100	O4'	OS	-0.369100
21	C1'	CT	-0.381261	C1'	CT	-0.450958	C1'	CT	-0.137381
22	H1'	H2	0.183800	H1'	H2	0.174600	H1'	H2	0.196300
23	N9	N*	-0.026800	N9	N*	0.057700	N1	N*	-0.033900
24	C8	C1	0.160700	C8	CK	0.073600	C6	CM	-0.018300
25	H8	H5	0.187700	H8	H5	0.199700	H6	H4	0.229300
26	N7	NB	-0.617500	N7	NB	-0.572500	C5	CM	-0.522200
27	C5	CB	0.072500	C5	CB	0.199100	H5	HA	0.186300
28	C6	CA	0.689700	C6	C	0.491800	C4	CA	0.843900
29	N6	N2	-0.912300	O6	O	-0.569900	N4	N2	-0.977300
30	H61	H	0.416700	N1	NA	-0.505300	H41	H	0.431400
31	H62	H	0.416700	H1	H	0.352000	H42	H	0.431400
32	N1	NC	-0.762400	C2	CA	0.743200	N3	NC	-0.774800
33	C2	CQ	0.571600	N2	N2	-0.923000	C2	C	0.795900
34	H2	H5	0.059800	H21	H	0.423500	O2	O	-0.654800
35	N3	NC	-0.741700	H22	H	0.423500	-	-	-
-36	C4	CB	0.380000	N3	NC	-0.663600	-	-	-
37	-	-	-	C4	CB	0.181400	-	-	-

Supplementary Table S2. Detail parameters of 2’-OCH_3 modified nonstandard nucleotides (DTO, GO5 and GO3) in the established force field of DTA_OMe.

	DTO			GO5			GO3		
**Serial number**	**Atom name**	**Atom type**	**Atom charge**	**Atom name**	**Atom type**	**Atom charge**	**Atom name**	**Atom type**	**Atom charge**
1	OP1	O2	-0.776100	N2	N2	-0.923000	OP1	O2	-0.776100
2	P	P	1.165900	H21	H	0.423500	P	P	1.165900
3	OP2	O2	-0.776100	H22	H	0.423500	OP2	O2	-0.776100
4	O5’	OS	-0.495400	C2	CA	0.743200	O5’	OS	-0.495400
5	C5’	CI	-0.006900	N3	NC	-0.663600	C5’	CI	-0.006900
6	H5’	H1	0.075400	C4	CB	0.181400	H5’	H1	0.075400
7	H5”	H1	0.075400	N1	NA	-0.505300	H5”	H1	0.075400
8	C4’	CT	0.162900	H1	H	0.352000	C4’	CT	0.162900
9	H4’	H1	0.117600	C6	C	0.491800	H4’	H1	0.117600
10	C3’	CE	0.071300	O6	O	-0.569900	C3’	CE	0.071300
11	H3’	H1	0.098500	C5	CB	0.199100	H3’	H1	0.098500
12	C2’	CT	0.944391	N7	NB	-0.572500	C2’	CT	1.047946
13	H2’	H1	0.472814	C8	CK	0.073600	H2’	H1	0.490953
14	O2’	OS	-1.229256	H8	H5	0.199700	O2’	OS	-1.275964
15	CM2	CT	0.679657	N9	N*	0.057700	CM2	CT	0.809411
16	H12	H1	-0.137819	C1’	CT	-0.450958	H12	H1	-0.175796
17	H13	H1	-0.137819	H1’	H2	0.174600	H13	H1	-0.175796
18	H14	H1	-0.137819	O4’	OS	-0.369100	H14	H1	-0.175796
19	O3’	OS	-0.523200	C4’	CT	0.162900	O3’	OH	-0.654900
20	O4’	OS	-0.369100	C5’	CI	-0.006900	HO3’	HO	0.439600
21	C1’	CT	-0.327949	O5’	OH	-0.631800	O4’	OS	-0.369100
22	H1’	H2	0.180400	HO5’	HO	0.442200	C1’	CT	-0.450958
23	N1	N*	-0.023900	H5’	H1	0.075400	H1’	H2	0.174600
24	C6	C2	-0.220900	H5”	H1	0.075400	N9	N*	0.057700
25	H6	H4	0.260700	H4’	H1	0.117600	C8	CK	0.073600
26	C5	C2	0.002500	C3’	CE	0.071300	H8	H5	0.199700
27	C7	CT	-0.226900	H3’	H1	0.098500	N7	NB	-0.572500
28	H71	HC	0.077000	O3’	OS	-0.523200	C5	CB	0.199100
29	H72	HC	0.077000	C2’	CT	1.047946	C6	C	0.491800
30	H73	HC	0.077000	H2’	H1	0.490953	O6	O	-0.569900
31	C4	C	0.519400	O2’	OS	-1.275964	N1	NA	-0.505300
32	O4	O	-0.556300	CM2	CT	0.809411	H1	H	0.352000
33	N3	NA	-0.434000	H12	H1	-0.175796	C2	CA	0.743200
34	H3	H	0.342000	H13	H1	-0.175796	N2	N2	-0.923000
35	C2	C	0.567700	H14	H1	-0.175796	H21	H	0.423500
36	O2	O	-0.588100	-	-	-	H22	H	0.423500
37	-	-	-	-	-	-	N3	NC	-0.663600
38	-	-	-	-	-	-	C4	CB	0.181400

Supplementary Table S3. Simulation details of protein, DNA aptamer and protein-DNA complex models in solvent.

Simulation system	Time (ns)	Number of atoms	Number of water molecules	Number of ions	Minimum distance (solute and box)(Å)
DEK_N	40	1857	5598	13×Cl^-	10
DEK_C	40	1198	6417	3×Cl^-	10
DTA	400	1300	8502	40×Na^+	10
DTA_OMe	400	1464	8407	40×Na^+	10
DEK_N/DTA	200	3132	12830	27×Na^+	12
DEK_N/DTA_OMe	200	3308	14368	27×Na^+	12
DEK_C/DTA	200	2394	13597	37×Na^+	12
DEK_C/DTA_OMe	200	2570	13384	37×Na^+	12

Supplementary Table S4. Per-residue free energy decomposition of DEK_N/DTA and DEK_N/DTA_OMe complexes (only show the negative energy).

DEK_N/DTA		DEK_N/DTA_OMe	
**Residue**	**Energy (kcal/mol)**	**Residue**	**Energy (kcal/mol)**
Phe78	-4.29	Phe78	-3.56
Thr79	-0.65	Thr79	-0.05
Ile80	-2.12	Ile80	-1.27
Ala81	-0.17	Ala81	-0.36
Gln82	-0.49	Gln82	-0.74
Gly83	-0.02	Gly83	-0.01
Lys84	-2.60	Lys84	-4.82
Lys87	-2.94	Gly85	-0.05
Leu88	-0.13	Lys87	-2.57
Cys89	-0.06	Leu88	-0.04
Arg93	-2.06	Cys89	-0.02
Hip95	-2.36	Arg93	-1.96
Phe97	-0.04	Hip95	-2.15
Leu98	-0.03	Lys100	-2.18
Lys100	-2.51	Lys101	-3.24
Lys101	-3.38	Lys102	-2.84
Lys102	-3.08	Thr103	-0.02
Arg107	-10.38	Arg107	-6.02
Asn108	-1.48	Asn108	-0.17
Leu109	-0.16	Hid110	-0.15
Hid110	-0.17	Lys111	-4.62
Lys111	-10.91	Leu112	-1.92
Leu112	-0.91	Tyr114	-1.23
Leu113	-0.10	Asn115	-0.95
Tyr114	-0.29	Arg116	-11.31
Asn115	-2.29	Pro117	-2.33
Arg116	-9.44	Gly118	-0.63
Pro117	-1.83	Thr119	-1.01
Gly118	-0.28	Val120	-0.81
Thr119	-0.08	Ser121	-0.13
Ser121	-0.18	Ser122	-1.00
Leu123	-0.02	Leu123	-0.16
Lys124	-3.03	Lys124	-2.44
Lys125	-7.75	Lys125	-2.65

Supplementary Table S4

DEK_N/DTA		DEK_N/DTA_OMe	
**Residue**	**Energy (kcal/mol)**	**Residue**	**Energy (kcal/mol)**
Asn126	-0.07	Asn126	-0.39
Val127	-0.13	Val127	-0.06
Gly128	-0.38	Gly128	-0.04
Gln129	-4.16	Gln129	-0.11
Phe130	-0.40	Phe130	-0.14
Ser131	-0.14	Phe135	-0.32
Phe133	-0.33	Lys137	-2.57
Pro134	-0.27	Ser139	-0.28
Phe135	-0.34	Val140	-3.72
Lys137	-2.48	Gln141	-2.41
Gly138	-0.04	Tyr142	-0.16
Ser139	-0.16	Lys143	-4.44
Val140	-2.06	Lys144	-8.74
Gln141	-1.40	Lys145	-3.96
Tyr142	-0.03	Met148	-0.28
Lys143	-3.38	Leu149	-0.11
Lys144	-5.60	Lys150	-3.16
Lys145	-4.39	Lys151	-3.36
Met148	-1.41	Phe152	-0.08
Leu149	-0.23	Arg153	-2.36
Lys150	-3.24	Lys158	-1.86
Lys151	-3.92	Val163	-0.03
Phe152	-3.66	Leu164	-0.03
Arg153	-7.26	Leu166	-0.02
Asn154	-2.32	Arg168	-2.00
Ala155	-1.51	Lys177	-2.13
Met156	-0.39	Arg178	-1.97
Leu157	-0.07	Hid185	-0.05
Lys158	-3.22	Pro186	-0.02
Ser159	-0.13	-	-
Ile160	-0.13	-	-
Cys161	-0.15	-	-
Val163	-0.11	-	-
Leu164	-0.08	-	-
Leu166	-0.03	-	-

Supplementary Table S4

DEK_N/DTA		DEK_N/DTA_OMe	
**Residue**	**Energy (kcal/mol)**	**Residue**	**Energy (kcal/mol)**
Arg168	-4.10	-	-
Ser169	-0.18	-	-
Val171	-0.04	-	-
Asn172	-0.07	-	-
Ser173	-0.01	-	-
Leu175	-0.07	-	-
Lys177	-2.58	-	-
Arg178	-2.23	-	-
Ile179	-0.03	-	-
Phe182	-0.04	-	-
Hid185	-0.06	-	-
Pro186	-0.06	-	-

Supplementary Table S5. Per-residue free energy decomposition of DEK_C/DTA and DEK_C/DTA_OMe complexes (only show the negative energy).

DEK_C/DTA		DEK_C/DTA_OMe	
**Residue**	**Energy (kcal/mol)**	**Residue**	**Energy (kcal/mol)**
Lys314	-11.46	Lys314	-13.32
Lys315	-8.16	Lys315	-4.93
Leu316	-0.29	Leu316	-1.11
Lys317	-13.25	Lys317	-7.95
Lys318	-7.94	Lys318	-4.84
Pro319	-0.07	Pro319	-0.30
Pro320	-1.57	Pro320	-0.17
Leu325	-0.19	Lys326	-2.80
Lys326	-2.60	Lys330	-3.02
Thr328	-0.12	Lys331	-4.05
Ile329	-0.02	Leu332	-0.65
Lys330	-3.13	Leu333	-0.06
Lys331	-5.84	Ala334	-0.14
Leu332	-1.44	Ser335	-0.16
Leu333	-0.16	Ala336	-0.16
Ala334	-0.43	Asn337	-0.08
Ser335	-0.35	Leu338	-0.05
Ala336	-0.23	Val341	-0.16
Asn337	-0.01	Lys344	-2.12
Leu338	-0.06	Gln345	-0.07
Val341	-0.15	Lys348	-2.62
Lys344	-2.19	Lys349	-8.99
Gln345	-0.02	Val350	-0.02
Lys348	-2.94	Tyr351	-0.03
Lys349	-3.88	Tyr354	-0.32
Asn353	-3.19	Pro355	-0.30
Tyr354	-1.94	Thr356	-0.31
Pro355	-2.88	Tyr357	-1.24
Tyr357	-1.93	Leu359	-0.09
Leu359	-0.18	Thr360	-0.11
Thr360	-0.04	Arg362	-2.87
Arg362	-2.48	Lys363	-2.54
Lys363	-2.37	Phe365	-0.04
Phe365	-0.02	Ile366	-0.03

Supplementary Table S5

DEK_C/DTA		DEK_C/DTA_OMe	
**Residue**	**Energy (kcal/mol)**	**Residue**	**Energy (kcal/mol)**
Ile366	-0.04	Lys367	-2.27
Lys367	-2.17	Thr368	-0.04
Thr368	-0.03	Thr369	-0.04
Thr369	-0.04	Val370	-0.02
Val370	-0.03	Lys371	-2.10
Lys371	-1.98	Leu373	-0.04
Leu373	-0.04	Ile374	-0.01
Ile374	-0.01	Ser375	-0.02
Ser375	-0.03	Leu376	-0.03
Leu376	-0.04	-	-

Supplementary Table S6. Detailed information of hydrogen bonds in DEK_N/DTA complex.

H-bond acceptor	DonorH	H-bond donor	Occupancy (%)	Average distance (Å) (acceptor-donor)
Ile80@O	DT10@H3	DT10@N3	63.74	2.84
DA40@OP2	Arg153@HH12	Arg153@NH1	63.67	2.79
DA40@OP2	Asn154@HD21	Asn154@ND2	62.13	2.84
DG3@OP2	Arg107@HH22	Arg107@NH2	52.41	2.82
DT10@OP1	Gln129@HE22	Gln129@NE2	52.18	2.82
DA40@OP1	Arg153@HH22	Arg153@NH2	49.99	2.83
DG3@OP2	Arg107@HH12	Arg107@NH1	41.48	2.82
DG2@OP1	Arg107@HH21	Arg107@NH2	39.34	2.82
DA34@OP2	Arg116@HH12	Arg116@NH1	35.31	2.80
DA34@OP2	Arg116@HH22	Arg116@NH2	34.53	2.81
DC35@O2	Lys111@HZ3	Lys111@NZ	32.88	2.76
DG2@OP1	Lys111@HZ1	Lys111@NZ	26.05	2.80
DC35@O2	Lys111@HZ1	Lys111@NZ	25.40	2.77
DT33@OP1	Lys144@HZ1	Lys144@NZ	25.18	2.79
DT33@OP1	Lys144@HZ3	Lys144@NZ	24.62	2.79
DT33@OP1	Lys144@HZ2	Lys144@NZ	23.43	2.80
DC35@O2	Lys111@HZ2	Lys111@NZ	22.92	2.77
Asn115@O	DC35@H41	DC35@N4	22.68	2.87
DA37@OP1	Asn108@HD21	Asn108@ND2	22.62	2.86
DT33@OP2	Asn115@HD22	Asn115@ND2	22.12	2.82
DA9@OP2	Lys125@HZ1	Lys125@NZ	19.86	2.79
DA9@OP2	Lys125@HZ2	Lys125@NZ	19.43	2.79
DA9@OP2	Lys125@HZ3	Lys125@NZ	19.33	2.79
DG2@OP1	Lys111@HZ2	Lys111@NZ	19.22	2.79
DA36@O3'	Asn108@HD21	Asn108@ND2	18.40	2.89
DA34@OP1	Asn115@HD21	Asn115@ND2	16.37	2.85
DG2@OP1	Lys111@HZ3	Lys111@NZ	16.25	2.80
DT10@O2	Ile80@H	Ile80@N	15.69	2.87
DT12@OP2	Phe78@H2	Phe78@N	13.44	2.79
DA11@OP2	Lys125@HZ1	Lys125@NZ	10.88	2.79
DT10@OP2	Gln129@HE22	Gln129@NE2	10.48	2.86
DG3@OP2	Arg107@HH21	Arg107@NH2	10.35	2.80

Supplementary Table S7. Detailed information of hydrogen bonds in DEK_N/DTA_OMe complex.

**H-bond acceptor**	**DonorH**	**H-bond donor**	**Occupancy (%)**	**Average distance (Å) (acceptor-donor)**
DCO27@OP1	Arg116@HH22	Arg116@NH2	87.66	2.81
DGO22@OP2	Arg107@HH12	Arg107@NH1	62.88	2.82
DGO22@OP2	Arg107@HH22	Arg107@NH2	61.69	2.83
DCO27@OP1	Arg116@HH12	Arg116@NH1	35.14	2.86
DGO26@O2'	Ser122@HG	Ser122@OG	29.49	2.60
DGO28@O4'	Thr79@HG1	Thr79@OG1	29.35	2.78
DCO16@OP1	Gln141@HE21	Gln141@NE2	26.47	2.85
DCO24@OP1	Ser121@HG	Ser121@OG	17.34	2.70
DTO21@OP1	Lys111@HZ1	Lys111@NZ	15.88	2.78
DTO21@OP1	Lys111@HZ3	Lys111@NZ	15.08	2.79
DTO21@OP1	Lys111@HZ2	Lys111@NZ	14.86	2.79
Gln82@OE1	DCO29@H42	DCO29@N4	14.47	2.86
DGO26@O3'	Arg116@HH12	Arg116@NH1	13.85	2.87
DGO22@O5'	Arg107@HH22	Arg107@NH2	13.67	2.88
DCO30@OP2	Asn115@HD22	Asn115@ND2	12.03	2.82
DCO16@OP1	Gln141@HE22	Gln141@NE2	11.93	2.84
DGO3@O4'	Lys144@HZ1	Lys144@NZ	11.71	2.84
DGO3@O4'	Lys144@HZ3	Lys144@NZ	11.42	2.84
DGO3@O4'	Lys144@HZ2	Lys144@NZ	11.31	2.84
DCO24@OP1	Thr119@HG1	Thr119@OG1	11.04	2.79
Asn115@OD1	DCO30@H42	DCO30@N4	10.41	2.84

Supplementary Table S8. Detailed information of hydrogen bonds in DEK_C/DTA complex.

H-bond acceptor	DonorH	H-bond donor	Occupancy (%)	Average distance (Å) (acceptor-donor)
DC29@OP1	Lys318@H	Lys318@N	59.27	2.84
Thr321@O	DC29@H42	DC29@N4	45.27	2.86
DC23@OP1	Ser335@HG	Ser335@OG	41.10	2.68
Asn353@OD1	DG22@H21	DG22@N2	38.47	2.85
Asn353@OD1	DG22@H1	DG22@N1	34.71	2.85
Glu327@OE1	DG26@H1	DG26@N1	19.49	2.81
DT5@OP1	Lys315@H	Lys315@N	15.18	2.84
DG28@OP1	Lys318@HZ1	Lys318@NZ	15.16	2.78
DC30@OP2	Lys317@HZ2	Lys317@NZ	14.65	2.81
DC29@OP2	Lys317@HZ3	Lys317@NZ	14.31	2.83
Glu327@OE2	DG26@H21	DG26@N2	13.78	2.85
DG28@OP1	Lys318@HZ3	Lys318@NZ	13.10	2.78
DC30@OP2	Lys317@HZ1	Lys317@NZ	12.55	2.81
DC29@OP2	Lys317@HZ2	Lys317@NZ	11.91	2.83
DC30@OP2	Lys317@HZ3	Lys317@NZ	11.84	2.81
DC29@OP2	Lys317@HZ1	Lys317@NZ	11.57	2.83
DG28@OP1	Lys318@HZ2	Lys318@NZ	11.16	2.78

Supplementary Table S9. Detailed information of hydrogen bonds in DEK_C/DTA_OMe complex.

H-bond acceptor	DonorH	H-bond donor	Occupancy (%)	Average distance (Å) (acceptor-donor)
DTO10@O2'	Lys349@HZ3	Lys349@NZ	27.59	2.73
DTO10@O2'	Lys349@HZ2	Lys349@NZ	27.38	2.73
DTO10@O2'	Lys349@HZ1	Lys349@NZ	26.02	2.73
DCO27@O2	Lys315@H	Lys315@N	24.05	2.86
DCO14@OP1	Lys314@H3	Lys314@N	20.43	2.81
DCO14@OP1	Lys314@H1	Lys314@N	19.47	2.80
DCO29@O4'	Thr356@HG1	Thr356@OG1	15.67	2.80
DCO30@OP2	Lys317@HZ3	Lys317@NZ	15.46	2.77
DCO14@OP1	Lys314@H2	Lys314@N	15.29	2.80
DCO30@OP2	Lys317@HZ1	Lys317@NZ	14.89	2.77
DCO15@OP2	Lys314@H2	Lys314@N	13.53	2.78
DAO11@OP1	Ser335@HG	Ser335@OG	13.50	2.68
DCO15@OP2	Lys314@H3	Lys314@N	12.94	2.78
DCO15@OP2	Lys314@H1	Lys314@N	11.70	2.79
DCO30@OP2	Lys317@HZ2	Lys317@NZ	11.67	2.77

Supplementary Table S10. Sequences and modification schemes of mutants (the cyan boxes are mutation sites and red letters are nucleotides modified with 2′-OCH_3 in the deoxyribose sugar unit).

Mutant	Aptamer sequence (5' to 3')
DTA	GGG GTT AAA TAT TCC CAC ATT GCC TGC GCC AGT ACA AAT AG
DT10DA	GGG GTT AAA AAT TCC CAC ATT GCC TGC GCC AGT ACA AAT AG
DT10DC	GGG GTT AAA CAT TCC CAC ATT GCC TGC GCC AGT ACA AAT AG
DT10DG	GGG GTT AAA GAT TCC CAC ATT GCC TGC GCC AGT ACA AAT AG
DC35DA	GGG GTT AAA TAT TCC CAC ATT GCC TGC GCC AGT AAA AAT AG
DC35DG	GGG GTT AAA TAT TCC CAC ATT GCC TGC GCC AGT AGA AAT AG
DC35DT	GGG GTT AAA TAT TCC CAC ATT GCC TGC GCC AGT ATA AAT AG
DCO29DAO	GGG GTT AAA TAT TCC CAC ATT GCC TGC GAC AGT ACA AAT AG
DCO29DGO	GGG GTT AAA TAT TCC CAC ATT GCC TGC GGC AGT ACA AAT AG
DCO29DTO	GGG GTT AAA TAT TCC CAC ATT GCC TGC GTC AGT ACA AAT AG
DCO30DAO	GGG GTT AAA TAT TCC CAC ATT GCC TGC GCA AGT ACA AAT AG
DCO30DGO	GGG GTT AAA TAT TCC CAC ATT GCC TGC GCG AGT ACA AAT AG
DCO30DTO	GGG GTT AAA TAT TCC CAC ATT GCC TGC GCT AGT ACA AAT AG
DG22DA	GGG GTT AAA TAT TCC CAC ATT ACC TGC GCC AGT ACA AAT AG
DG22DC	GGG GTT AAA TAT TCC CAC ATT CCC TGC GCC AGT ACA AAT AG
DG22DT	GGG GTT AAA TAT TCC CAC ATT TCC TGC GCC AGT ACA AAT AG
DG26DA	GGG GTT AAA TAT TCC CAC ATT GCC TAC GCC AGT ACA AAT AG
DG26DC	GGG GTT AAA TAT TCC CAC ATT GCC TCC GCC AGT ACA AAT AG
DG26DT	GGG GTT AAA TAT TCC CAC ATT GCC TTC GCC AGT ACA AAT AG
DC29DA	GGG GTT AAA TAT TCC CAC ATT GCC TGC GAC AGT ACA AAT AG
DC29DG	GGG GTT AAA TAT TCC CAC ATT GCC TGC GGC AGT ACA AAT AG
DC29DT	GGG GTT AAA TAT TCC CAC ATT GCC TGC GTC AGT ACA AAT AG
DCO27DAO	GGG GTT AAA TAT TCC CAC ATT GCC TGA GCC AGT ACA AAT AG
DCO27DGO	GGG GTT AAA TAT TCC CAC ATT GCC TGG GCC AGT ACA AAT AG
DCO27DTO	GGG GTT AAA TAT TCC CAC ATT GCC TGT GCC AGT ACA AAT AG
